# Galectin-8 drives ERK-dependent mitochondrial fragmentation, perinuclear relocation and mitophagy, with metabolic adaptations for cell proliferation

**DOI:** 10.1016/j.ejcb.2025.151488

**Published:** 2025-06

**Authors:** Adely de la Peña, Claudio Retamal, Francisca Pérez-Molina, Nicole Díaz-Valdivia, Francisco Veloso-Bahamondes, Diego Tapia, Jorge Cancino, Felix Randow, Alfonso González, Claudia Oyanadel, Andrea Soza

**Affiliations:** a Centro de Biología Celular y Biomedicina, CEBICEM, Facultad de Ciencias, Universidad San Sebastián, Santiago, Chile; b Escuela de Medicina, Facultad de Medicina, Universidad San Sebastián, Santiago, Chile; c Departamento de Ciencias Biológicas y Químicas, Facultad de Ciencias, Universidad San Sebastián, Santiago, Chile; d Division of Protein and Nucleic Acid Chemistry, MRC Laboratory of Molecular Biology, Cambridge, UK; e Department of Medicine, University of Cambridge, UK; f Centro Científico Tecnológico de Excelencia Ciencia y Vida, Fundación Ciencia y Vida, Santiago, Chile

**Keywords:** Galectin-8, Mitochondrial dynamics, Proliferation, Mitophagy, Glycosylation

## Abstract

Mitochondria adapt to the cell proliferative demands induced by growth factors through dynamic changes in morphology, distribution, and metabolic activity. Galectin-8 (Gal-8), a carbohydrate-binding protein that promotes cell proliferation by transactivating the EGFR-ERK signaling pathway, is overexpressed in several cancers. However, its impact on mitochondrial dynamics during cell proliferation remains unknown. Using MDCK and RPTEC kidney epithelial cells, we demonstrate that Gal-8 induces mitochondrial fragmentation and perinuclear redistribution. Additionally, mitochondria adopt donut-shaped morphologies, and live-cell imaging with two Keima-based reporters demonstrates Gal-8-induced mitophagy. ERK signaling inhibition abrogates all these Gal-8-induced mitochondrial changes and cell proliferation. Studies with established mutant versions of Gal-8 and CHO cells reveal that mitochondrial changes and proliferative response require interactions between the N-terminal carbohydrate recognition domain of Gal-8 and α-2,3-sialylated N-glycans at the cell surface. DRP1, a key regulator of mitochondrial fission, becomes phosphorylated in MDCK cells or overexpressed in RPTEC cells in an ERK-dependent manner, mediating mitochondrial fragmentation and perinuclear redistribution. Bafilomycin A abrogates Gal-8-induced cell proliferation, suggesting that mitophagy serves as an adaptation to cell proliferation demands. Functional analysis under Gal-8 stimulation shows that mitochondria maintain an active electron transport chain, partially uncoupled from ATP synthesis, and an increased membrane potential, indicative of healthy mitochondria. Meanwhile, the cells exhibit increased extracellular acidification rate and lactate production via aerobic glycolysis, a hallmark of an active proliferative state. Our findings integrate mitochondrial dynamics with metabolic adaptations during Gal-8-induced cell proliferation, with potential implications for physiology, disease, and therapeutic strategies.

## Highlights

• Galectin-8 induces mitochondrial fragmentation and perinuclear redistribution.
• Galectin-8 enhances mitophagy, specifically in a subset of mitochondria.
• Galectin-8 modulates mitochondrial dynamics via carbohydrate recognition-ERK-DRP1.
• Galectin-8 increases the extracellular acidification rate and lactate production via aerobic glycolysis.

## 1 Introduction

Mitochondria are versatile organelles with bioenergetic, biosynthetic, and signaling roles essential for integrating extracellular stimuli and intracellular metabolic adaptations, thereby coordinating responses to physiological demands such as cell proliferation (Jenkins et al., 2024, Li et al., 2024, Meacham et al., 2022, Vander Heiden and DeBerardinis, 2017). The best-known regulatory pathway linking cell proliferation with mitochondrial adaptations is the canonical system mediated by growth factors (Lopez-Mejia and Fajas, 2015, Vyas et al., 2016). Growth factors bind and activate specific cell surface receptors, which initiate intracellular signaling cascades that lead to synchronized mitochondrial and cell cycle changes (Lopez-Mejia and Fajas, 2015, Mitra et al., 2009, Vyas et al., 2016). Cancer cells exploit this integration between mitochondrial function and growth factor signaling to sustain continuous proliferation and enhance survival (Deepak et al., 2024, Li et al., 2024, Suomalainen and Nunnari, 2024, Vyas et al., 2016). Less attention, however, has been given to alternative regulatory proteins, including the galectin family of carbohydrate-binding proteins that contribute to deciphering the information embedded in the “sugar code” of glycoproteins and glycolipids, thereby modifying cellular functions (Barake et al., 2020, Cerliani et al., 2017, Dennis et al., 2009, Johannes et al., 2018, Nabi et al., 2015, Troncoso et al., 2023).

Mitochondrial function is tightly connected to mitochondrial dynamics, encompassing changes in structure, subcellular distribution, and functional status, which adapt mitochondria to metabolic demands in response to extracellular signals (Friedman and Nunnari, 2014, Jenkins et al., 2024, Suomalainen and Nunnari, 2024). During cell proliferation, metabolic changes often include increased glucose utilization via fermentation, resulting in lactate production and secretion, even in the presence of oxygen (Jenkins et al., 2024, Li et al., 2024, Vander Heiden and DeBerardinis, 2017). This process, known as aerobic glycolysis or the Warburg effect in cancer cells, reflects mitochondrial adaptations that limit ATP generation via oxidative phosphorylation while maintaining oxygen consumption in a partially uncoupled state prone to generating metabolites for cell growth (Jenkins et al., 2024, Li et al., 2024, Martinez-Reyes and Chandel, 2021). Mitochondrial dynamics also control the generation of ROS and ATP through oxidative phosphorylation (OXPHOS) coupled with oxygen consumption (Huang et al., 2023, Picard and Shirihai, 2022). Fission is driven by Dynamin-related/-like protein 1 (DRP1) (Breitzig et al., 2018), and fusion is mediated by mitofusin 1 and 2 (MFN1 and MFN2) in the outer mitochondrial membrane (OMM) and optic atrophy 1 (OPA1) in the inner mitochondrial membrane (IMM) (Tilokani et al., 2018). Mitochondrial dynamics also include movements along microtubules and actin filaments mediated by specific motor and anchoring proteins (Furnish and Caino, 2020), as well as the selective degradation of damaged and superfluous mitochondria through mitophagy (Chen and Chan, 2017, Rolland et al., 2013). Mitochondrial dynamics respond to extracellular signals, facilitating cellular adaptations to environmental and physiological demands (Picard and Shirihai, 2022), including cell proliferation and differentiation (Jenkins et al., 2024, Suomalainen and Nunnari, 2024). Dysregulation of these processes is implicated in numerous diseases, including cancer (Suomalainen and Nunnari, 2024, Vyas et al., 2016).

Galectins modulate a broad spectrum of cellular processes through selective binding to β-galactoside-containing glycans on various cell surface glycoproteins and glycolipids (Barake et al., 2020, Cerliani et al., 2017, Johannes et al., 2018, Nabi et al., 2015, Rabinovich et al., 2012). As galectins lack a signal peptide for the exocytic pathway, they play intracellular roles in the cytosol and extracellular roles after their unconventional secretion (Barake et al., 2020, Perez-Moreno et al., 2024). A distinctive feature of this regulatory system is that structural variations within β-galactosides configure a “sugar code” sensitive to physiological and pathological changes, which dynamically influences galectin binding affinity and function (Cerliani et al., 2017, Dennis et al., 2009, Rabinovich et al., 2012).

Gal-8 is a widely expressed galectin in human tissues and cancer cells (Cagnoni et al., 2020), which promotes cell proliferation (Oyanadel et al., 2018, Shatz-Azoulay et al., 2020, Zick, 2022) and is overexpressed in several cancers, often correlating with a poor prognosis (Elola et al., 2014). As a tandem repeat galectin, Gal-8 is comprised of two carbohydrate recognition domains (CRD) separated by a linker peptide segment of isoform-specific length (Brewer et al., 2002). A unique feature of Gal-8 among other galectins is its preference for terminal α-2,3-sialic acid provided by its N-terminal CRD (Cagnoni et al., 2020), while its C-terminal CRD, similar to other galectins, exhibits affinity for non-sialylated β-galactosides (Elola et al., 2014). Intracellularly, Gal-8 participates in a protective surveillance system that detects damaged endo-lysosomes and bacteria-containing phagosomes to facilitate their autophagic removal (Hoyer et al., 2022, Jia et al., 2019, Jia et al., 2020, Thurston et al., 2016, Thurston et al., 2012). After its unconventional secretion, extracellular Gal-8 acts as an autocrine stimulus of cell proliferation, primarily interacting with selected β1-integrins on the cell surface (Carcamo et al., 2006, Elola et al., 2014, Perez-Moreno et al., 2024, Zick, 2022). Our previous studies in non-tumorigenic MDCK cells showed that extracellular Gal-8, either in an autocrine manner following secretion from overexpressing transfected cells or when externally added as recombinant protein, transactivates EGFR downstream of β1-integrin-associated FAK kinase, promoting cell proliferation and migration via the ERK signaling pathway (Oyanadel et al., 2018). Such autocrine signaling may similarly drive proliferation of cancer cells overexpressing Gal-8 (Elola et al., 2014). On a related note, recent evidence showed that Gal-8 can be used to stimulate epithelial cell proliferation and differentiation, along with other protective effects against acute kidney injury, suggesting therapeutic potential (Perez-Moreno, Toledo, et al., 2024).

Here, we investigate whether Gal-8 induces changes in mitochondrial dynamics accompanying its stimulation of cell proliferation. Our results reveal that Gal-8 promotes fragmentation and perinuclear redistribution of mitochondria in epithelial cells through interactions with α-2,3 sialylated N-glycans and ERK-DRP1 activation. Additionally, Gal-8 enhances mitophagy and modifies metabolic activities, resulting in decreased ATP linked to oxygen consumption and increased extracellular acidification rate, indicating a shift towards aerobic glycolysis associated with cell proliferation. Mitochondria maintain their health as assessed by their increased membrane potential. These findings establish Gal-8 as a novel regulator of mitochondrial dynamics, with potential implications for both physiological and pathological processes.

## 2 Methods

### 2.1 Antibodies and Reagents

Monoclonal and polyclonal antibodies from Cell Signaling Technology: DRP1 (#8570), pS616-DRP1 (#3455), p44/42-ERK (#4695), Phospho-p44/42-ERK (#4370), Ki67 (#9129) and GAPDH (#2118); HRP-conjugated secondary antibodies (Dako); Phalloidin (A30104 Thermo Fisher Scientific); Hoechst (34580 Thermo Fisher Scientific); Bovine serum albumin (BSA) powder, glycerol, glycine and methanol (Thermo Fisher Scientific), HEPES, Tris-HCL, Trizma base and glutathione (Sigma-Aldrich) Tween 20 and Triton X-100 (VWR), dithiothreitol (DTT) (Melford) distilled water (Life Technologies); Fetal bovine serum (FBS) (Labtech); Penicillin Streptomycin - P/S, Glutamine, IPTG (Isopropyl β-D-1-thiogalactopyranoside), paraformaldehyde (Sigma-Aldrich); Protease inhibitors including leupeptin, aprotinin, benzamide and phenylmethylsulphonyl fluoride (PMSF) (Sigma-Aldrich); Agents for cell selection: Ampicillin (Melford), puromycin and Blasticidin S HCl (Life Technologies), kanamycin (Sigma-Aldrich). PD98059 (513001 - Sigma-Aldrich); Bafilomycin A (Sigma-Aldrich); Cytochalasin D (Sigma-Aldrich); MitoTracker™ Orange CMTMRos (Cat. M7510; Thermo Fisher Scientific).

### 2.2 Cell lines and culture conditions

HEK293ET, CHO-K1-WT, and CHO-LEC3.2.8.1 cells (American Type Culture Collection), RPTEC cells (Sigma-Aldrich), and MDCK cells (gift from Dr. Chiara Zurzolo, Pasteur Institute, Paris, France). HEK293ET, CHO-K1-WT, and CHO-LEC3.2.8.1, and MDCK cells were grown in DMEM (Dulbecco's Modified Eagle Medium; Thermo Fisher Scientific) containing 10 % FBS and P/S. RPTEC cells were grown in MEM-α (Minimum Essential Medium; Thermo Fisher Scientific) supplemented with RPTEC complete supplement (Sigma-Aldrich), P/S, and 2 mM Glutamine. All cell lines were routinely tested for mycoplasma contamination.

### 2.3 Generation of Gal-8 specific carbohydrate-binding mutants

Primers pairs complementary to human Gal-8: 5’-ACATGTCCATGATGTTGTCCTTAAACAACC-3’ and 3-’GCGGCCGCCTACCAGCTCCTTACTTCCAGTAAGTG-5’ were used to amplify the Gal-8 sequence by PCR from cDNA-GST-Gal-8 plasmid constructs. Assembly PCR was used to introduce point mutations (Zeng et al., 2018). Primers for site-directed mutagenesis of Gal-8 at position 69: 5’-GGCAGCAGCATGAAACCT GCC GCCGATGTGGCCTTTCAT −3’ and 3’-ATGAAAGGCCACATCGGC GGC AGGTTTCATGCTGCTGCC-5’, and at the position 275: 5’-GCTCTACACTTGAACCCA CAT CTGAATATTAAAGCATTT-3’ and 3’-AAATGCTTTAATATTCAG ATG TGGGTTCAAGTGTAGAGC-5’.

### 2.4 Production of recombinant galectins

pETM30 vector was used to express proteins fused to His-GST in *E. coli* strain BL21, as described by Ravenhill (Ravenhill et al., 2019). After transfection with plasmid constructs encoding Gal-8 or its mutants, bacteria were grown to an OD_600_ of 0.6–0.8, and protein expression was induced with 0.1 mM IPTG (Isopropyl β-D-1-thiogalactopyranoside) for 16 hours at 16°C. The bacterial pellets, harvested at 200 rpm, were resuspended in bacterial lysis buffer (20 mM Tris-HCl (pH 7.4), 150 mM NaCl, 1 mM DTT, 1 tablet of complete EDTA-free Protease inhibitor cocktail (Roche) per 50 ml, and 20 μg/ml DNaseI (Sigma-Aldrich) for 60 min at 4°C and sonicated at 100 Hz. Bacterial lysates were centrifuged at 20000 rpm, and the supernatants (cleared lysate) were added to a column of GST-Sepharose (GE Healthcare) for 2 hours at 4 °C, then washed the column and eluted the protein with glutathione buffer (20 mM Tris-HCl (pH 7.4), 150 mM NaCl, 1 mM DTT, and 20 mM Glutathione (pH 8.0). The eluate was incubated with TEV protease (1 %) overnight at 4°C. To remove the GST, the purified proteins were incubated with Ni-NTA magnetic agarose beads in 10 mM Imidazole. Protein expression was confirmed by SDS-PAGE followed by Coomassie Blue staining technique (Figure S1).

### 2.5 Virus production

Retrovirus-containing supernatants were generated in HEK293ET cells as outlined by Ravenhill (Ravenhill et al., 2019). Briefly, HEK293ET cells were plated at 1 × 10^5^ cells per well in a 6-well plate. A transfection mix was prepared containing 1 μg of M6Pblast-Keima-FIS1, M6Pblast-MT-Keima, M6Pblast-MT-GFP or sh-DRP1 (Addgene), 500 ng of a proviral plasmid, and plasmids for the VSV-G envelope and retroviral gag/pol, along with 0.1 mg/ml Polyethylenimine (PEI - Sigma-Aldrich). After 48 hours at 37°C, the virus-containing supernatants were collected and centrifuged at 5000 rpm for 3 minutes. For transduction, 500 μl of the viral supernatants were added to MDCK or RPTEC cells. Selection of drug-resistant retrovirus-transduced cells was performed using the appropriate antibiotic 48 hours post-transduction.

### 2.6 Western blot

Cells were lysed in Lysis Buffer (20 mM Tris pH 7.4, 150 mM NaCl, 1.0 % Triton X-100) with protease and phosphatase inhibitors. The lysates were separated on 4 %–12 % denaturing gels and transferred to pre-equilibrated Immobilon-P PVDF membranes. Membranes were incubated with primary antibodies followed by HRP-conjugated secondary antibodies. Protein detection was performed using ECL reagents, and the membranes were developed with an iBright 1500 system. Band intensity was quantified using ImageJ.

### 2.7 Proliferation assay

Cells (1 ×10^4^) were seeded onto glass coverslips, serum-starved by removing FBS and then treated under the following conditions: A) Pre-treatment with PD98059 (25 μM) or Bafilomycin A (100 nM) for 30 minutes before adding Gal-8 (50 µg/ml) for 24 hours, maintaining the PD98059 and Bafilomycin A during the entire period; B) Treatment with Gal-8, Gal-8-R69H, Gal-8-R275H, Gal-8-R69H-R275H, or Gal-4 (50 μg/ml) for 24 hours, fixed with 4 % paraformaldehyde in PBS, and incubated for 1 hour at 37°C with anti-Ki67 and then nuclear-stained with Hoechst (1 ng/ml). Coverslips were mounted onto glass slides using Fluoromount-G antifade reagent and allowed to dry for 24 hours. Samples were analyzed with an Olympus FV1200 confocal microscope using a 20X objective. Approximately 900 nuclei were counted and compared to the number of Ki67-positive nuclei to determine the percentage of Ki67-positive cells. ImageJ was used for counting and quantification.

### 2.8 Mitochondria morphology analysis

Cells (1 ×10^4^) were seeded and cultured for 24 hours on glass coverslips. They were serum-starved by removing FBS and then treated under the following conditions: A) Pre-treatment with PD98059 (25 μM) or Cytochalasin D (2 μM) for 30 minutes before adding Gal-8 (50 µg/ml) to the medium for 24 hours, maintaining the inhibitors throughout this period. B) Treatment with Gal-8, Gal-8-R69H, Gal-8-R275H, Gal-8-R69H-R275H, Gal-1, Gal-3, or Gal-4 (50 μg/ml), or CCCP (10 μM) for 24 hours. Mitochondria were stained with MitoTracker Orange CMTMRos (500 ng/ml) for 30 minutes, fixed with 4 % paraformaldehyde in PBS, and rinsed with distilled water containing Hoechst (1 ng/ml) to stain the nuclei. The coverslips were then mounted on glass slides using Fluoromount-G antifade reagent and allowed to dry for 24 hours. Samples were analyzed with a Leica TCS SP8 confocal microscope using a 63X oil immersion lens.

### 2.9 Substrate coating

Glass-bottomed Petri dishes were coated with 50 μg/ml Gal-8 and/or 25 μg/ml Fibronectin (Sigma-Aldrich, #F2006), or with PBS as a control, for 2 hours at room temperature in PBS, and then washed five times with PBS. Afterward, MDCK-MT-GFP cells (1 ×10^4^) were seeded onto the protein-coated cover glass in DMEM without serum for 24 hours. The cells were subsequently fixed with 4 % paraformaldehyde in PBS. Actin filaments were stained using phalloidin, and the nucleus was stained with Hoechst (1 ng/ml). Samples were analyzed using a Leica TCS SP8 confocal microscope with a 63X oil immersion lens.

### 2.10 Live Cell Imaging

To measure mitophagy, FIS1-Keima or MT-Keima transfected cells (1 ×10^4^ cells) were cultured on LabTek 35 mm glass bottom dishes, serum-starved by depleting FBS, and then treated under the following conditions: A) Pre-treatment with PD98059 (25 μM) for 30 minutes before adding Gal-8 (50 µg/ml) to the medium for 24 hours, maintaining PD98059 throughout the entire period. B) Treatment with Gal-8, Gal-8-R69H, Gal-8-R275H, or Gal-8-R69H-R275H (50 μg/ml), or CCCP (10 μM) for 24 hours. Live cells were imaged in DMEM-HEPES at 37ºC using a Leica TCS SP8 confocal microscope with a 63X oil immersion lens. The fluorescence of FIS1-Keima or MT-Keima was captured in two channels via two sequential excitations (458 nm and 561 nm lasers), detecting emissions from 570 to 695 nm.

To measure mitochondrial membrane potential, MDCK-MT-GFP cells (1 ×10^4^ cells) were seeded in LabTek 35 mm glass bottom dishes and treated with either vehicle or Gal-8 (50 µg/ml) for 24 hours, followed by incubation with tetramethylrhodamine, ethyl ester – TMRE (0.5 μM) for 30 minutes at 37ºC in the dark. After three washes with PBS, the cells were maintained in DMEM HEPES at 37ºC for live-cell imaging on a Leica TCS SP8 confocal microscope using a 63X oil immersion lens. The laser intensity and gain were held constant across all conditions for comparative analysis.

### 2.11 Seahorse XFp assay

Oxygen Consumption Rate (OCR) and Extracellular Acidification Rate (ECAR) of live MDCK cells were measured using the Seahorse XFp system (Agilent) as described by Anderson (Anderson et al., 2018). In brief, 20000 cells were plated per well were treated with either vehicle or Gal-8 (50 μg/ml) for 24 hours at 37°C. On the analysis day, assay media (25 mM glucose, 1 mM sodium pyruvate, and 4 mM L-glutamine) were prepared and adjusted to pH 7.4. The cells were then equilibrated in a non-CO2 incubator for 60 minutes. The XFp cartridge was loaded with Oligomycin (100 μM), FCCP (100 μM), and a mixture of Rotenone and Antimycin A (50 μM) for the Cell Mito Stress test. The OCR and ECAR results were normalized to control basal levels to account for experimental variability. Data were collected from four independent experiments, each with three replicates.

### 2.12 Lactate assay

Lactate levels were measured using a Lactate Assay Kit from BioVision Inc/Abcam (Cat #ab65330) following the manufacturer’s protocol. Briefly, lactate was detected from cells seeded at approximately 60–70 % confluence in a 6-well plate. The cells were treated without FBS, using either a vehicle or Gal-8 (50 μg/ml) for 24 hours, then trypsinized and lysed in 100 μl of ice-cold PBS through three cycles of flash freeze-thaw. Cell debris was removed by centrifugation at 1000 rpm for 4 minutes, and the remaining supernatant was collected for analysis. A 20 μl volume of supernatant was added to white opaque 96-well plates along with 30 μl of lactate assay buffer, followed by 50 μl of assay buffer plus enzyme mix. A negative control lacking enzymes, a positive control, and a standard curve were employed to calculate the lactate concentration. All samples were incubated for 30 minutes at room temperature in the dark. Optical density at 570 nm or fluorescence at Ex/Em = 544/590 nm was measured using a BioTek Synergy HTX Multimode Reader. Sample concentration calculations were performed as described in the manufacturer’s protocol.

### 2.13 Software analysis

Huygens Essential software (SVI, Netherlands) was used to restore and deconvolve confocal images. The particle analyzer plugin was used to segment and quantify the number, length, surface area, and distance between objects (mitochondria-nucleus).

### 2.14 Statistics analysis

All data analysis was performed in GraphPad Prism 8. Results were plotted as mean ± standard deviation (s.d.). Statistical significance was assessed based on the experiment: a Student's *t*-test was used for comparisons between two samples, and ANOVA followed by Tukey's post hoc test was used for comparisons among more than two samples. For non-parametric data, the Kruskal–Wallis test was used, followed by Dunn's test to define the differences between each sample. P-values of P < 0.05 (*), P < 0.01 (**), and P < 0.001 (***), considered statistically significant, are indicated in each figure. No statistical difference was indicated as ns (not significant).

## 3 Results

### 3.1 Galectin-8 induces fragmentation and redistribution of mitochondria to the perinuclear zone in a carbohydrate-dependent manner associated with cell proliferation

We previously reported that MDCK cells respond to extracellular Gal-8 by increasing proliferation through a FAK-EGFR-ERK signaling pathway in a carbohydrate-binding manner (Oyanadel et al., 2018). Mitochondrial dynamics are essential for cell proliferation, ensuring an even distribution of this metabolically crucial organelle between daughter cells (Lawrence et al., 2016, Rohn et al., 2014). We therefore asked whether Gal-8 influences mitochondrial dynamics through the same carbohydrate-binding mechanism that mediate the proliferative effect of Gal-8 on MDCK cells.

**Fig. 1:**
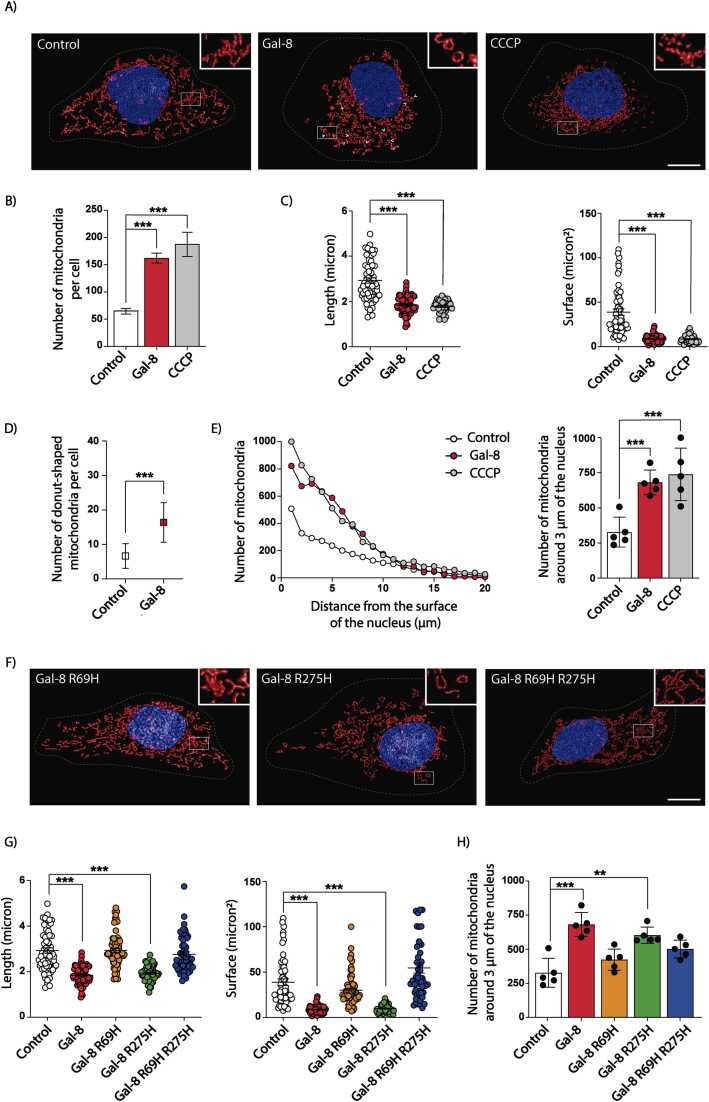
**Gal-8 induces mitochondrial fragmentation and redistribution in a carbohydrate-dependent manner**. MDCK cells were treated with Gal-8, Gal-8-R69H, Gal-8-R275H, Gal-8-R69H-R275H (50 µg/ml), or CCCP (10 μM) for 24 hours and then incubated with MitoTracker CMTMRos to visualize mitochondria. Images were acquired using a confocal microscope and Z-stacks were deconvolved in 3D, surface rendered, and analyzed with Huygens Essential software. A) Representative images of control, Gal-8 and CCCP treated cells. Graphs show the following mitochondrial characteristics: B) number, C) length and surface, D) number of donut-shaped mitochondria (indicated by white arrows), E) distribution within the cell based on the distance from the nuclear perimeter. F) Representative images of mitochondria in control, Gal-8, Gal-8-R69H, Gal-8-R275H, Gal-8-R69H-R275H treated cells. G) and H) graphs show the indicated quantification and the statistical significance in each condition (Mean ± s.d., n = 3, 25 cells per experiment, One-Way ANOVA with a posterior Tukey, ***p < 0.001). Scale bar = 10μm.

Treatment with Gal-8 (50 μg/ml) for 24 hours increased the number of mitochondria, as revealed by MitoTracker CMTMRos staining (Fig. 1A-B). Mitochondria exhibited reduced length and surface area, consistent with mitochondrial fragmentation, thus resembling the effects of CCCP, a well-known inducer of mitochondrial fragmentation (Kwon et al., 2017) (Fig. 1C). However, unlike CCCP, Gal-8 also induced a distinctive donut-shaped mitochondrial morphology (representative image in Fig. 1A and D). Additionally, mitochondria redistributed toward the perinuclear region (Fig. 1E), with the majority accumulating within 3 µm of the nucleus (Fig. 1E). CHO-K1 and RPTEC cells exhibited similar mitochondrial changes following Gal-8 treatment (Figure S2A-E). These results indicate that Gal-8 promotes fragmentation, perinuclear redistribution, and morphological changes in mitochondria, including the formation of donut-shaped organelles.

### 3.2 Galectin-8 promotes mitochondrial fragmentation and perinuclear distribution through interactions with cell surface sialylated glycans

**Fig. 2:**
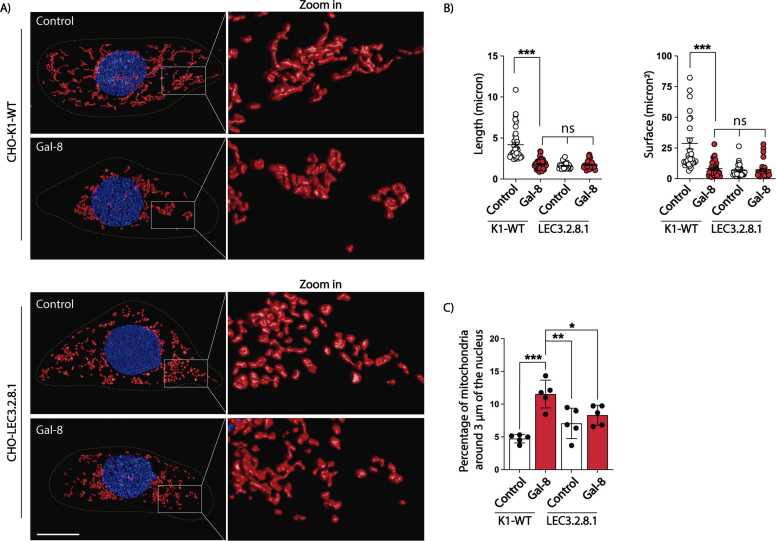
Gal-8-induced fragmentation and perinuclear distribution of mitochondria require surface sialylated glycoconjugates. CHO-K1-WT and CHO-LEC3.2.8.1 cells were treated with Gal-8 (50 μg/ml) for 24 hours and then labeled with MitoTracker CMTMRos and Hoechst. Z-stacks of confocal images were deconvolved, surface rendered, and analyzed with Huygens Essential software. A) Representative images of CHO-K1-WT and CHO-LEC3.2.8.1 cells. B) Graphs showing the length and surface of mitochondria. C) Percentage of mitochondria within 3 μm of the nucleus. (Mean ± s.d., n = 3, 25 cells per experiment, One-Way ANOVA with a posterior Tukey, *p < 0.05, **p < 0.01, ***p < 0.001). Scale bar = 10μm.

To evaluate whether the effects of Gal-8 on mitochondrial fragmentation and perinuclear redistribution are mediated through glycan interactions, we used Gal-8 alleles specifically impaired in their ability to bind glycans. Gal-8-R69H lacks carbohydrate-binding activity in its N-terminal CRD, preventing interactions with sialylated β-galactosides. Gal-8-R275H disrupts binding to polylactosamine galactosides, while the double mutant Gal-8-R69H-R275H is unable to interact with both sialylated β-galactosides and polylactosamine galactosides (Hirabayashi et al., 2002). Gal-8-R69H and the double mutant Gal-8-R69H-R275H both failed to induce mitochondrial fragmentation or perinuclear redistribution in MDCK (Fig. 1F-H) and CHO-K1 (Figure S3) cells. In contrast, the Gal-8-R275H mutant, which retains binding to sialylated β-galactosides, fully replicated the mitochondrial fragmentation and perinuclear relocation effects observed with wild-type Gal-8 (Fig. 1F-H and S3). To further assess the role of sialylated glycans in Gal-8’s effects on mitochondria, we used CHO-LEC3.2.8.1 cells, which are deficient in sialylation (Patnaik and Stanley, 2006, Stanley, 1989). Under basal conditions, CHO-LEC3.2.8.1 cells already exhibited mitochondrial fragmentation, although without perinuclear distribution, compared to their wild-type counterparts, CHO-K1 cells (Fig. 2). Gal-8 treatment did not affect the widespread cytosolic distribution of mitochondria in these cells (Fig. 2C). We conclude that Gal-8 promotes mitochondrial fragmentation and perinuclear distribution by interacting with cell surface α-2,3 sialylated glycoproteins via its N-terminal CRD domain.

### 3.3 Selective effects of galectin-8 on mitochondrial dynamics among galectin family members

**Fig. 3:**
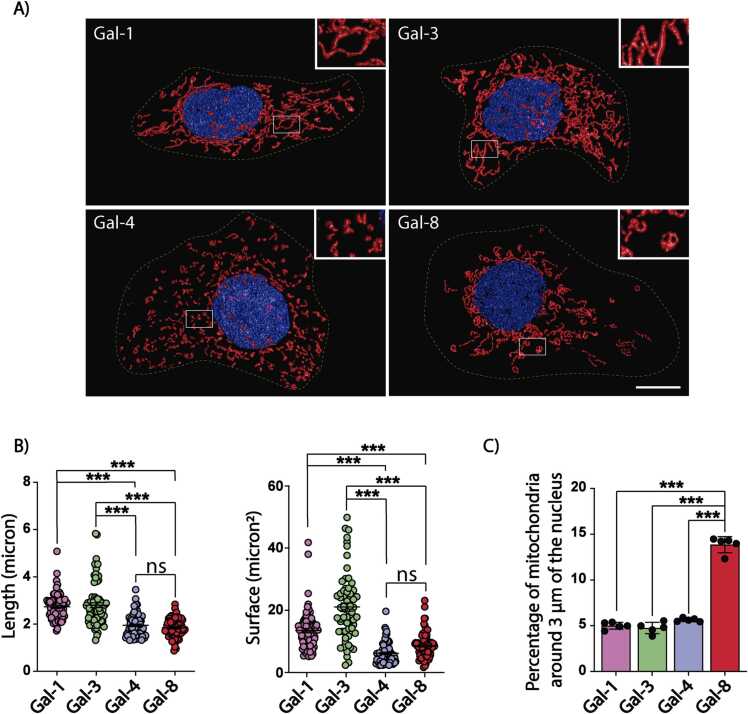
Comparing the effects of Gal-8 with other galectins on mitochondrial fragmentation and redistribution MDCK cells were treated with Gal-1, −3, −4, or −8 (50 μg/ml) for 24 hours were then labeled with MitoTracker CMTMRos and images were acquired by confocal microscopy. Z-stacks, deconvolved, surface rendered, and analyzed with Huygens Essential software. A) Representative images of MDCK cells after each treatment. B) Graphs showing the length and surface of mitochondria. C) Percentage of mitochondria within 3 μm of the nucleus. (Mean ± s.d., n = 3, 25 cells per experiment, One-Way ANOVA with a posterior Tukey, ***p < 0.001). Scale bar = 10μm.

To assess the selectivity of Gal-8’s effects, we tested other members of the galectin family, including prototype (Gal-1), tandem repeat (Gal-4), and chimeric-only (Gal-3) types (Johannes et al., 2018). Both tandem repeat galectins, Gal-4 and Gal-8, reduced mitochondrial length and surface area, whereas neither Gal-1 nor Gal-3 elicited these effects (Fig. 3A-B). Amongst the tested galectin, only Gal-8, induced the perinuclear redistribution of mitochondria (Fig. 3C).

### 3.4 Galectin-8-driven mitochondrial remodeling correlates with cell proliferation in an ERK-dependent manner

**Fig. 4:**
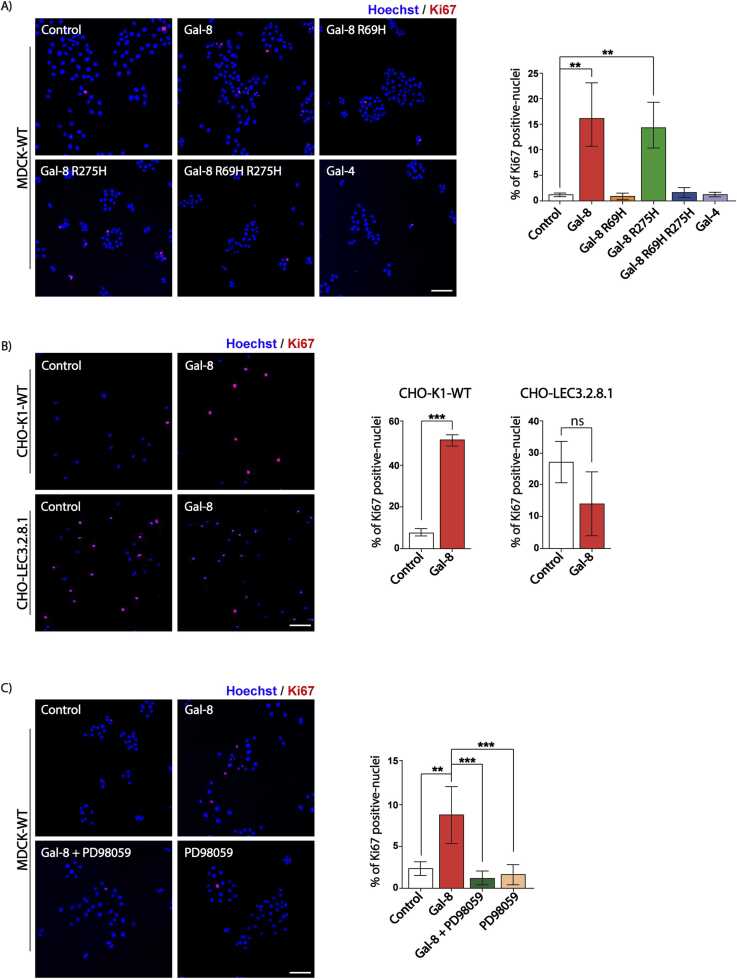
Gal-8 promotes cell proliferation in a carbohydrate- and ERK-dependent manner. Ki67 positive cell rate in A) MDCK cells treated with Gal-8, Gal-8-R69H, Gal-8-R275H, Gal-8-R69H-R275H or Gal-4 (50 µg/ml) for 24 hours, B) CHO-K1-WT and CHO-LEC3.2.8.1 cells treated with Gal-8 (50 μg/ml) for 24 hours, and C) MDCK cells treated with Gal-8 (50 μg/ml) in the presence or absence of MEK inhibitor PD98059 (25 μM) for 24 hours. (Mean ± s.d., n = 3, 300 cells per experiment, One-Way ANOVA with a posterior Tukey, *p < 0.05, **p < 0.01, ***p < 0.001). Scale bar = 100μm.

These results allowed us to evaluate whether Gal-8’s effects on mitochondrial dynamics correlate with its described role in epithelial cell proliferation (Oyanadel et al., 2018). We evaluated Ki67 expression, a proliferation marker, in MDCK cells treated for 24 hours with either wild-type or mutant Gal-8, under the same conditions used for mitochondrial dynamics analysis (Fig. 4A). Only Gal-8 and Gal-8-R275H induced MDCK cell proliferation, whereas Gal-8-R69H had no effect (Fig. 4A). Similarly, RPTEC cells exhibited increased proliferation in response to Gal-8 (Figure S2F). Interestingly, although Gal-4 mimicked Gal-8’s effects on mitochondrial fragmentation without inducing perinuclear redistribution (Fig. 3), it did not promote cell proliferation (Fig. 4A). Additionally, CHO-LEC3.2.8.1 cells, which showed higher basal proliferation than CHO-K1 cells, did not exhibit a Gal-8-induced increase in proliferation, unlike wild-type CHO-K1 cells (Fig. 4B).

**Fig. 5:**
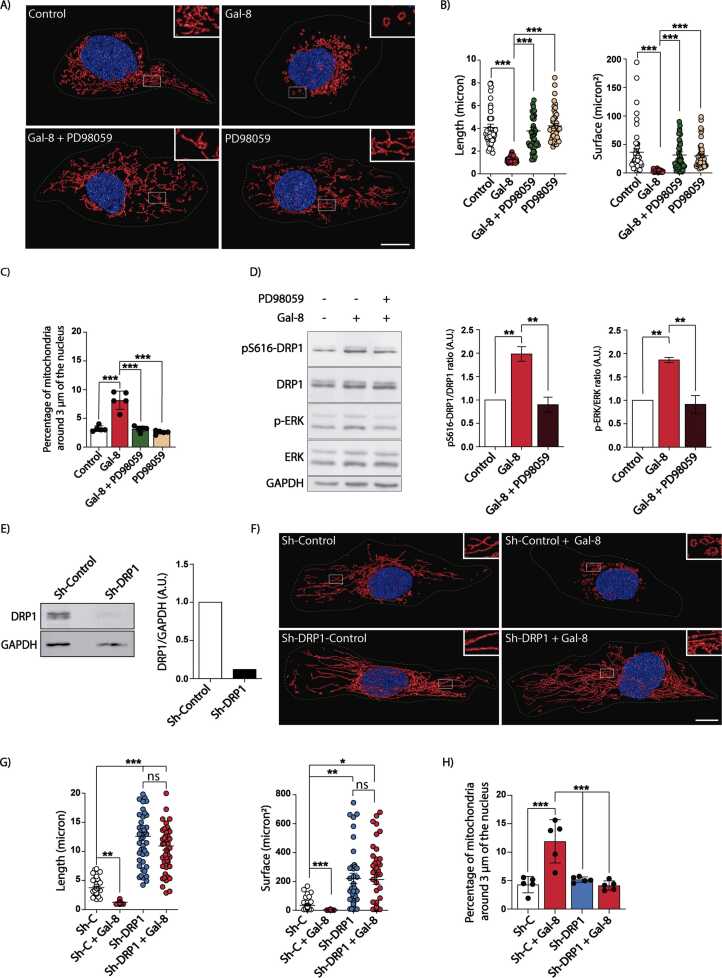
Gal-8-induced fragmentation and redistribution of mitochondria to the perinuclear zone depend on ERK-mediated DRP1 phosphorylation. MDCK cells treated with Gal-8 (50 μg/ml) in the presence or absence of MEK inhibitor PD98059 (25 μM) for 24 hours were stained with MitoTracker CMTMRos and Hoechst. Z-stacks of images acquired by confocal microscopy were deconvolved, surface rendered and analyzed with Huygens Essential software. A) Representative images. B) Graphs showing the length and surface of mitochondria. C) Percentage of mitochondria within 3 μm of the nucleus. D) Immunoblots of pS616-DRP1, DRP1, pERK, and ERK of MDCK cells treated as indicated. The graphs show the increase in the phosphorylation rate relative to the control. E) Immunoblot shows the level of DRP1 in RPTEC-Sh-Control and RPTEC-Sh-DRP1. F) Representative images of RPTEC-Sh-Control and RPTEC-Sh-DRP1 in the presence or absence of Gal-8. G) Graphs showing the length and surface of mitochondria of RPTEC-Sh-Control and RPTEC-Sh-DRP1. H) Percentage of mitochondria within 3 μm of the nucleus of RPTEC-Sh-Control and RPTEC-Sh-DRP1 in the presence or absence of Gal-8. (Mean ± s.d., n = 3, 15 cells per experiment, One-Way ANOVA with a posterior Tukey or Kruskal–Wallis with a posterior Dunn's test *p < 0.05, **p < 0.01, ***p < 0.001). Scale bar = 10μm.

To determine whether Gal-8-induced mitochondrial changes and cell proliferation share a common ERK signaling dependency, we pre-treated the cells with the MEK1 inhibitor PD98059 for 30 min before Gal-8 stimulation. MEK inhibition abolished both Gal-8-induced cell proliferation, as measured by Ki67 staining (Fig. 4C) as well as fragmentation and perinuclear redistribution of mitochondria (Fig. 5A-C).

These findings strongly suggest that Gal-8’s effects on mitochondrial dynamics and cell proliferation are interconnected through interactions with cell surface glycoproteins and downstream ERK signaling.

### 3.5 Galectin-8-induced mitochondrial fragmentation involves ERK-mediated DRP1 regulation

Known ERK substrates include key regulators of mitochondrial dynamics (Prieto et al., 2016, Pyakurel et al., 2015, Sessions and Kashatus, 2021). Among them, DRP1 is a central regulator of mitochondrial fission (Chen et al., 2023, Ko et al., 2016). Depending on the cellular context, ERK phosphorylates DRP1 at S616, enhancing its activity and promoting mitochondrial fission at the site where it binds to the outer membrane (Chen et al., 2023, Ko et al., 2016). Therefore, we investigated whether DRP1 mediates Gal-8-induced mitochondrial changes.

In MDCK cells, Gal-8 treatment for 24 hours significantly increased DRP1 phosphorylation at S616, except when cells were pre-treated for 30 min with a MEK inhibitor (Fig. 5D). In RPTEC cells, MEK inhibition also prevented Gal-8-induced mitochondrial fragmentation and perinuclear redistribution (Figure S4A-C), mirroring its effects in MDCK cells. However, unlike in MDCK cells, RPTEC cells did not show increased DRP1 phosphorylation in response to Gal-8. Instead, these cells increased their DRP1 protein levels in an ERK-dependent manner (Figure S4D), consistent with previous reports linking elevated DRP1 expression to enhanced mitochondrial fission (Adebayo et al., 2021).

Since mitochondrial fission can also occur independently of DRP1 (Che et al., 2015, Pagliuso et al., 2018), we silenced DRP1 in RPTEC cells via lentiviral infection (Fig. 5E). DRP1-knockdown RPTEC cells (RPTEC-Sh-DRP1) exhibited a more interconnected mitochondria network than control cells and did not undergo mitochondrial fragmentation or perinuclear redistribution following Gal-8 treatment (Fig. 5F-H).

We conclude that Gal-8 activates an ERK-dependent pathway that regulates DRP1 function to drive mitochondrial fragmentation and subsequent perinuclear distribution.

### 3.6 Galectin-8 induces mitophagy

Mitochondrial fragmentation is often linked to mitochondrial turnover through mitophagy, a selective autophagic process that removes damaged, old, or dysfunctional mitochondria to maintain cellular health and function (Sun et al., 2017). To assess mitophagy, we transfected MDCK cells with an expression plasmid for FIS1 or MT mitochondrial proteins fused to Keima, a pH-sensitive dual-excitation ratiometric fluorescent protein resistant to lysosomal proteases (Albornoz et al., 2024, Sun et al., 2017). Mitophagy was analyzed by live cell imaging based on Keima fluorescence shift from 440 nm at neutral (pH 7), to 586 nm in the acidic lysosomal environment (pH 4.5) (Albornoz et al., 2024, Sun et al., 2017).

**Fig. 6:**
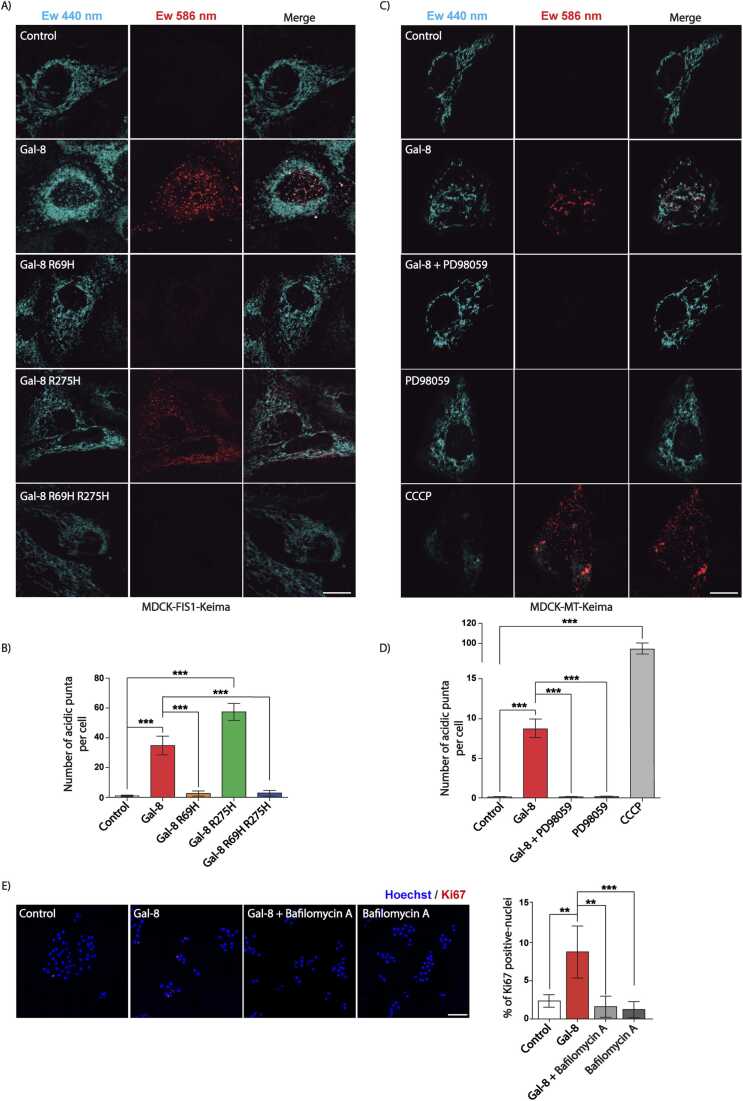
Gal-8 promotes mitophagy in carbohydrate- and ERK activity-dependent manners impacting on cell proliferation. A) Live-cell imaging confocal microscopy of MDCK-FIS1-Keima cells treated with Gal-8, Gal-8-R69H, Gal-8-R275H, or Gal-8-R69H-R275H (50 µg/ml) for 24 hours. B) Graph showing the quantification of mitochondria in mitophagy (red). C) Live-cell imaging confocal microscopy of MDCK-MT-Keima treated with Gal-8 (50 μg/ml) in the presence or absence of MEK inhibitor PD98059 (25 μM) for 24 hours, using CCCP as inducer of mitophagy. (D) Graph showing the quantification of mitochondria in mitophagy (red). E) Ki67 positive cell rate in MDCK cells treated with Gal-8 (50 μg/ml) in the presence or absence of Bafilomycin A (100 nM) for 24 hours. (Mean ± s.d., n = 3, 15 cells per experiment for mitophagy, 300 cells per experiment for proliferation, One-Way ANOVA with a posterior Tukey, **p < 0.01, ***p < 0.001). Scale bar = 10μm for A-C) and 100μm for E).

Following Gal-8 treatment, confocal live-cell imaging revealed mitochondrial accumulation of both FIS1-Keima (Fig. 6A-B) and MT-Keima (Fig. 6C-D) in lysosomes, reflecting mitophagy. The greater number of acidic puncta observed with FIS1-Keima compared with MT-Keima likely reflects differences in their sub-mitochondrial location. FIS1, an outer mitochondrial membrane protein (Ihenacho et al., 2021), may be more readily exposed to the acidic lysosomal pH than MT-Keima, which is targeted to the mitochondrial matrix (Sun et al., 2017).

### 3.7 Galectin-8-induced mitophagy is linked to mitochondrial fragmentation and cell proliferation

In MT-Keima expressing cells, we also tested CCCP, a well-established mitophagy inducer (Koncha et al., 2021), and observed the expected increase in acidic puncta (Fig. 6C-D). Similarly, Gal-8-R275H induced mitophagy, whereas Gal-8-R69H or Gal-8-R69H-R275H mutants had no significant effect (Fig. 6A-B). Inhibition of the ERK pathway with a MEK inhibitor abrogated Gal-8-induced mitophagy (Fig. 6C-D). These findings suggest that Gal-8 induces mitophagy through the same pathways that drives mitochondrial fragmentation and perinuclear redistribution, requiring interactions between of its N-CRD with α-2,3-sialylated glycans at the cell surface as well as ERK signaling. Our results align with previous reports linking mitophagy to mitochondrial fragmentation (Wakabayashi et al., 2009), which is mediated by Gal-8 through ERK signaling.

The relationship between mitophagy and cell proliferation has been mainly studied in tumor cells, where it can either promote or counteract proliferation depending on the cellular system and the stimulus causing mitophagy (Dong and Zhang, 2024). Enhanced mitophagy has been observed under conditions of active proliferation in tumor cells (Barra et al., 2024, Dong and Zhang, 2024, Mauro-Lizcano et al., 2024). In such cases, its inhibition by Bafilomycin A, a well-known inhibitor of autophagy, also inhibits cell proliferation (Mauvezin and Neufeld, 2015). Consistently, we found that Bafilomycin A not only reduced Gal-8-induced cell proliferation but also affected basal proliferation levels (Fig. 6E), as reported in other studies (Lu et al., 2015, Xie et al., 2014, Yan et al., 2016). These findings suggest that mitophagy-driven removal of damaged mitochondria is likely required to sustain active cell proliferation.

### 3.8 Galectin-8 affects mitochondrial metabolic function

Mitochondrial dynamics influence aerobic ATP production, with highly fused tubular mitochondrial networks correlating with higher ATP synthesis, whereas fragmented mitochondria are linked to reduced oxidative phosphorylation and elevated glycolytic rates (Chen and Chan, 2017, Chiu et al., 2021, Gomes et al., 2011, Jheng et al., 2012, Liu et al., 2020).

**Fig. 7:**
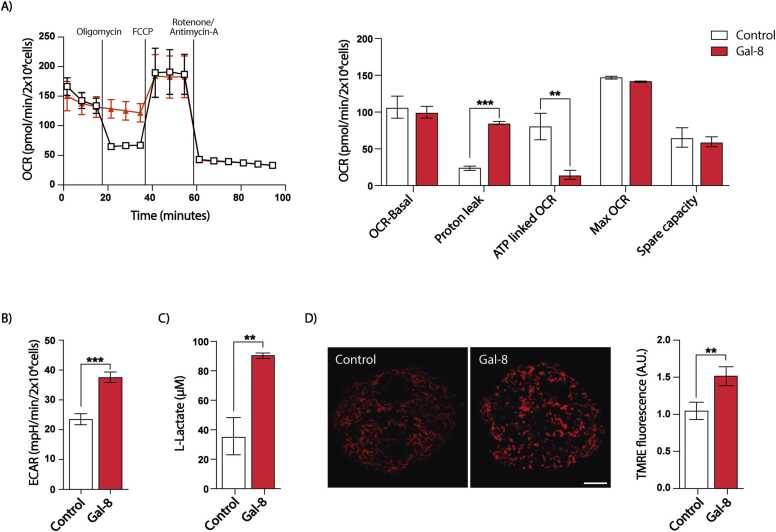
Gal-8 promotes a glycolytic state with no variation on the basal OCR. A) Profile of Seahorse XFp Cell Mito Stress Test assay in MDCK cells treated with vehicle or Gal-8 (50 µg/ml) for 24 hours. The graph shows the relative values of parameters in (A). B) Graph showing the basal ECAR measurement obtained using the Seahorse XFp assay. C) Lactate levels in MDCK cell lysates. D) TMRE fluorescence intensity in MDCK cells. (Mean ± s.d., n = 3 or 4, T-student, **p < 0.01, ***p < 0.001, One-Way ANOVA with a posterior Tukey, **p < 0.01, ***p < 0.001). Scale bar = 10μm.

To assess the metabolic impact of Gal-8 on mitochondria, we performed a Seahorse XFp assay, in which cells are sequentially treated with different mitochondrial inhibitors (Caines et al., 2022), including Oligomycin (ATP synthase inhibitor), FCCP (an uncoupling agent that dissipates the electrochemical hydrogen gradient), and Rotenone/Antimycin-A (two respiratory chain inhibitors), while glucose, sodium pyruvate, and L-glutamine were provided as substrates (Yoo et al., 2024). MDCK cells treated with Gal-8 for 24 hours exhibited reduced ATP production alongside increased proton leakage levels after Oligomycin treatment, whereas basal OCR, maximum OCR, and spare capacity remained unaffected (Fig. 7A). Gal-8 also increased the extracellular acidification rate (ECAR) (Fig. 7B), associated with elevated lactate levels (Fig. 7C) and indicative of enhanced aerobic glycolysis.

To further assess mitochondrial health, we measured mitochondrial membrane potential using TMRE, a fluorescent dye that accumulates in active mitochondria (Perry et al., 2011). Interestingly, Gal-8 treatment increased the mitochondrial membrane potential in MDCK cells (Fig. 7D), suggesting that mitochondria remain functionally competent despite undergoing fragmentation.

These results indicate that Gal-8-induced mitochondrial fragmentation and redistribution in epithelial cells are accompanied by metabolic adaptations, including partial uncoupling with reduced ATP production associated with oxygen consumption, increased mitochondrial membrane potential levels, and increased anaerobic glycolysis.

## 4 Discussion

This study establishes Gal-8 as an extracellular regulator of mitochondrial dynamics with metabolic implications for epithelial cell proliferation. Through interactions with α-2,3-sialylated N-glycans on the cell surface, Gal-8 promotes ERK-dependent mitochondrial fission, followed by perinuclear mitochondrial redistribution and mitophagy, leading to metabolic adaptations that sustain increased proliferative activity while maintaining mitochondrial health. Although Gal-8 is also known for intracellular functions within the cytosol (Hoyer et al., 2022, Jia et al., 2019, Jia et al., 2020, Thurston et al., 2016, Thurston et al., 2012), our findings emphasize its extracellular role in orchestrating mitochondrial remodeling during cell proliferation.

We show that Gal-8 added to the culture medium of MDCK cells elicits fragmentation and perinuclear distribution of mitochondria, coinciding with increased cell proliferation observed under the same conditions. Several findings strongly suggest that Gal-8’s effects on mitochondrial dynamics are intertwined with cell proliferation. Experiments using mutant versions of Gal-8 indicate that both mitochondrial dynamics and cell proliferation require interactions of this lectin with α-2,3-sialylated β-galactosides at the cell surface. A Gal-8 mutant lacking β-galactoside binding in the C-terminal CRD but retaining an intact N-terminal CRD (Thurston et al., 2016, Thurston et al., 2012) remains competent in promoting mitochondrial fragmentation and perinuclear redistribution together with cell proliferation. In contrast, a Gal-8 mutant with disrupted N-terminal CRD binding to α-2,3-sialylated β-galactosides (Hirabayashi et al., 2002, Thurston et al., 2016) loses the ability to induce mitochondrial fragmentation and perinuclear relocation, as well as cell proliferation. Additionally, sialylation-deficient CHO-LEC3.2.8.1 cells (Patnaik and Stanley, 2006, Stanley, 1989), which already exhibit fragmented mitochondria, fail to respond to Gal-8 stimulation, neither relocating mitochondria to the perinuclear region nor increasing proliferation. Therefore, Gal-8 interactions with sialylated β-galactosides at the cell surface are the essential trigger for mitochondrial fragmentation, perinuclear relocation, and cell proliferation.

On the other hand, our previous studies in MDCK cells showed that Gal-8 induces FAK/EGFR/ERK signaling that promotes cell proliferation (Oyanadel et al., 2018). By adjusting the experimental conditions, we found that after 24 hours of Gal-8 treatment, both mitochondrial dynamics and cell proliferation were sensitive to MEK inhibition, revealing their shared dependency on ERK signaling. ERK-mediated phosphorylation of DRP1 at its S616 residue has been implicated in mitochondrial fission (Gan et al., 2014, Sessions and Kashatus, 2021, Yu et al., 2011), and a FAK-ERK pathway has been associated with mitochondrial fission through DRP1 phosphorylation (Chang et al., 2022). We show that Gal-8-induced ERK activation enhances DRP1 phosphorylation at S616 in MDCK cells and increases DRP1 protein levels in RPTEC cells, two DRP1 modifications that have been involved in mitochondrial fragmentation (Adebayo et al., 2021, Chang et al., 2022). Furthermore, DRP1-silencing prevents mitochondrial fragmentation in response to Gal-8, confirming its role in this process. These findings indicate that Gal-8’s effects on mitochondrial dynamics and cell proliferation rely on the same molecular requirements, involving interactions with sialylated β-galactosides at the cell surface and downstream ERK signaling. The most straightforward interpretation is that mitochondrial dynamics and cell proliferation are functionally interconnected during Gal-8 stimulation.

Mitochondrial fragmentation generates smaller, more mobile mitochondria, facilitating their transport along the cytoskeleton (Huang et al., 2023, Moore and Holzbaur, 2018, Sessions and Kashatus, 2021). However, the fragmented mitochondria seen in Gal-4-treated MDCK cells and sialylation-incompetent CHO-LEC3.2.8.1 cells lack perinuclear redistribution, indicating that mitochondrial fragmentation and relocation are not necessarily linked. Under Gal-8 stimulation, the perinuclear positioning of fragmented mitochondria likely involves cytoskeletal components, such as microtubules and/or actin filaments, along with motor and anchoring proteins on the outer mitochondrial membrane (Boldogh and Pon, 2007, Chen et al., 2023, Eberhardt et al., 2020, Flannery and Trushina, 2019, Furnish and Caino, 2020, Lovas and Wang, 2013). The specific mechanism elicited by Gal-8 remains to be defined. An interesting possibility for future studies is the role of filamin A, a regulator of actin filaments, in perinuclear mitochondrial clustering, as recently described in response to integrin-mediated extracellular matrix (ECM) stiffness (Daga et al., 2024). Gal-8 may trigger a similar mechanism, given its known ability to bind fibronectin, an ECM component (Smith et al., 2020), and integrins, which interact with actin filaments (Carcamo et al., 2006, Diskin et al., 2012, Vicuna et al., 2013).

The perinuclear distribution of mitochondria has been associated with various cellular functions (Daga et al., 2024, Jenkins et al., 2024). These include macromolecules transport across nuclear pores and delivering mitochondrial proteins (Naik et al., 2019), buffering nuclear calcium during cytoplasmic calcium fluctuations (Park et al., 2001), and regulating reactive oxygen species (ROS) as second messengers in transcriptional responses to hypoxia (Al-Mehdi et al., 2012). Additionally, perinuclear mitochondria contribute to oxygen level regulation within the nucleus through respiratory changes, influencing cellular processes and gene expression, and potentially contributing to oxidative stress and aging (Mori et al., 2023). Transcriptional regulation of gene expression by mitochondrial perinuclear gathering has been reported in response to ECM stiffness in MCF-7 epithelial cells, where this reorganization promotes the nuclear location of RUNX2 transcription factor that drives osteogenesis (Daga et al., 2024). Our results show that Gal-4, another tandem-repeat galectin similar to Gal-8 (Perez-Moreno, Oyanadel, et al., 2024), induces mitochondrial fragmentation without perinuclear redistribution and without affecting cell proliferation, demonstrating that mitochondrial fragmentation alone is insufficient to promote cell proliferation. The preference for sialic acid binding distinguishes Gal-8 from other galectins (Perez-Moreno, Oyanadel, et al., 2024) and likely explains its unique influence on mitochondrial dynamics. Mitochondria relocation to the perinuclear region in response to Gal-8, but not Gal-4, may send signals essential for cell proliferation into the nucleus, similar to those described in epithelial-mesenchymal transition (EMT) (Desai et al., 2020).

The cell proliferative state is typically accompanied by a metabolic shift, where mitochondria transition toward an anabolic mode to generate metabolites as building blocks, while ATP production predominantly relies on aerobic glycolysis (Suomalainen and Nunnari, 2024). Mitochondrial structure and cellular energy balance are tightly interconnected (Liesa and Shirihai, 2013). Fragmented mitochondria are generally linked to impaired oxidative phosphorylation (OXPHOS), leading to reduced ATP synthesis and increased ROS production (Chiu et al., 2021, Tabara et al., 2021, Westermann, 2012). We show that in Gal-8 treated cells, mitochondria maintain their basal oxygen consumption rate (OCR) even in the presence of the ATP synthase inhibitor Oligomycin. This suggests that the electron transport chain (ETC) remains active but is partially uncoupled from ATP synthesis, likely due to proton re-entry into the mitochondrial matrix through uncoupling proteins (UCPs) (Jastroch et al., 2010, Ledesma et al., 2002, Wolkow and Iser, 2006). Additionally, Gal-8-treated cells exhibit increased ECAR, elevated lactate levels, and a rise in mitochondrial membrane potential. The effect of Gal-8 on mitochondrial proton leak contrasts with the increase in mitochondrial membrane potential, suggesting a compensatory increase in electron transport chain activity (Divakaruni and Brand, 2011, Jastroch et al., 2010). Thus, Gal-8 acts as an external stimulus driving mitochondria toward a less efficient ATP production state, favoring glycolysis over oxidative phosphorylation. The increase in ECAR and lactate levels indicates a metabolic shift to glycolysis for energy production, converting glucose to lactate (Mookerjee et al., 2015). This Gal-8-stimulated metabolic reprogramming is characteristic of rapidly proliferating states, such as the Warburg effect in cancer cells, and may also contribute to reducing ROS production (Potter et al., 2016).

A notable feature accompanying the Gal-8 effects is the mitochondrial acquisition of a donut-like shape. The functional meaning of this mitochondrial phenotype remains unclear and may depend on the cellular context (Jenkins et al., 2024). Donut-shaped mitochondria have been observed under various conditions, including oxidative stress and intracellular calcium fluctuations (Ahmad et al., 2013), mitochondrial potential loss during hypoxia-reoxygenation (Liu and Hajnoczky, 2011), and metabolic stress (Lionetti et al., 2014). This morphology has also been associated with both pathological and physiological processes, appearing in conditions such as Alzheimer’s disease (Hara et al., 2014), cardiac damage (Lampert et al., 2019), and osteoblast differentiation (Suh et al., 2023). Interestingly, MCF-7 epithelial cells display donut-shaped mitochondria in response to ECM stiffness (Daga et al., 2024), suggesting a potential link between mitochondrial remodeling and mechanotransduction. Gal-8 interactions with integrins may mimic cellular responses to ECM components, as seen in lamellipodia formation (Carcamo et al., 2006). Donut-shaped mitochondria have been associated with interactions with the actin cytoskeleton (Chakrabarti et al., 2022). However, Gal-8 treatment may induce this morphology through a different mechanism, as it remains unaffected by Cytochalasin D treatment (Figure S5). Furthermore, the alterations in mitochondrial dynamics induced by Gal-8 are not induced by fibronectin (Figure S6), a glycoprotein of the extracellular matrix that binds to β1-integrins (Li et al., 2021). These findings indicate a more specific Gal-8-elicited mitochondrial changes, likely involving additional intracellular signaling mechanisms than those triggered by extracellular matrix components such as fibronectin (Jiang et al., 2002). Different effects of Gal-8 and fibronectin have been previously described regarding lamellipodia formation and spreading in Jurkat cells (Carcamo et al., 2006). Donut-shaped mitochondria have been proposed to enhance metabolic flexibility, allowing cells to adapt to stress and maintain mitochondrial function under challenging conditions, involving preservation of membrane potential, increased surface area for organelle contact, and resistance to autophagy (Jenkins et al., 2024, Liu and Hajnoczky, 2011, Long et al., 2015, Suh et al., 2023, Xue et al., 2020). Under Gal-8 stimulation, donut-shaped mitochondria may represent a population escaping enhanced mitophagy, serving as a mitochondrial reservoir adapted to meet the metabolic demands of increased cell proliferation.

Mitophagy, the selective removal of mitochondria by autophagy, is enhanced in response to Gal-8, as demonstrated by live-cell imaging with FIS1- and MT-Keima reporters. Observations using Gal-8 mutants and MEK inhibition indicate that the same pathways driving mitochondrial fragmentation and perinuclear redistribution are also involved in mitophagy. Specifically, Gal-8-induced mitophagy depends on interactions with α-2,3-sialylated glycans at the cell surface, followed by ERK signaling. Furthermore, mitophagy often requires prior mitochondrial fission to remove dysfunctional segments (Wakabayashi et al., 2009) and is frequently associated with mitochondrial positioning near the perinuclear region (Collins et al., 2002, Lacombe and Scorrano, 2024), two changes induced by Gal-8. The co-existence of mitophagy with healthy mitochondria, as indicated by increased membrane potential, suggests a pro-proliferative role of mitophagy, potentially coupled with mitochondria biogenesis. Indeed, we found that Bafilomycin A, used to inhibit autophagy and, therefore, also mitophagy, prevents Gal-8-induced cell proliferation, further supporting this link.

Several studies, primarily in cancer biology, have attributed either promoting (Barra et al., 2024, Chang et al., 2017, Mauro-Lizcano et al., 2024, Wang et al., 2023) or suppressive (Deng et al., 2023, Tang et al., 2023, Wang et al., 2022) roles to mitophagy in cell proliferation and tumor progression, depending on the cellular context and the nature of the mitophagic stimulus (Dong and Zhang, 2024). Mitophagy is critical in maintaining mitochondrial and cellular oxidative homeostasis and integrity by selectively removing dysfunctional, supernumerary, or aged mitochondria (Antico Arciuch et al., 2012, Clague and Urbe, 2025, Harper et al., 2018, Palikaras et al., 2018). During proliferation, cells regulate mitochondrial quantity and quality by balancing biogenesis and degradation, with mitophagy selectively removing damaged or dysfunctional mitochondria to maintain cellular homeostasis (Palikaras et al., 2018). Mitophagy can eliminate metabolically unsuited mitochondria while biogenesis replenishes the mitochondrial pool with newly generated, better-suited organelles (Drake and Yan, 2017, Palikaras et al., 2015a, Palikaras et al., 2015b, Palikaras et al., 2016, Palikaras et al., 2018, Palikaras and Tavernarakis, 2014). Mitophagy and mitochondrial biogenesis can be coordinated to drive metabolic reprogramming (Palikaras et al., 2015a, Palikaras et al., 2015b, Palikaras et al., 2016, Palikaras et al., 2018, Palikaras and Tavernarakis, 2014), favoring cell proliferation (Palikaras and Tavernarakis, 2014). Dysregulated mitophagy can lead to the development of various diseases and metabolic disorders (Miao et al., 2023, Tang et al., 2024). Therefore, Gal-8-induced mitophagy may contribute to mitochondrial quality control, ensuring a healthy mitochondrial network during cell proliferation. Two main pathways regulate mitophagy: the ubiquitin-dependent (PINK1-PRKN-dependent) pathway and the receptor-dependent (PINK1- PRKN-independent) pathway (Palikaras et al., 2018), each potentially exerting distinct biological functions. The sensitivity of these pathways to Gal-8 stimulation remains to be elucidated.

Interestingly, elevated BNIP3 levels in breast cancer cell lines (MCF7 and MDA-MB-231) have been linked to increased metabolic activity, cell proliferation, and migration (Mauro-Lizcano et al., 2024). Similarly, overexpression of Gal-8 in MDCK cells promotes an epithelial-mesenchymal transition (EMT), likely through autocrine stimulation after its secretion into the media, leading to cell proliferation, invasive migration, and tumorigenesis (Oyanadel et al., 2018). These observations suggest that Gal-8 overexpression, reported in several carcinomas (Elola et al., 2014), may contribute to cancer-adaptive mitophagic activity (Deepak et al., 2024, Lee et al., 2023). In a different context, Gal-8 has been shown to protect against acute kidney injury (AKI) induced by folic acid (Perez-Moreno, Toledo, et al., 2024), a known promoter of mitochondrial dysfunction (Aparicio-Trejo et al., 2020). Gal-8 not only favors the proliferation of epithelial cells during kidney repair but also reduces their death during the injury phase (Perez-Moreno, Toledo, et al., 2024). Gal-8-triggered mitochondrial fragmentation, perinuclear redistribution, and mitophagy may contribute to survival mechanisms in diseases involving mitochondrial dysfunction. Our findings position Gal-8 as an extracellular stimulus that integrates cell proliferation signaling with mitochondrial dynamics to mediate metabolic reprogramming.

The extracellular role of Gal-8 in regulating mitochondrial dynamics opens new control possibilities and therapeutic opportunities based on the structural changes that glycans undergo under physiological and pathological conditions (Cerliani et al., 2017, Dennis et al., 2009). Variations in N-glycan branching complexity occurring at the Golgi apparatus define different affinities for galectins (Dennis et al., 2009), and the Gal-8 N-terminal CRD preference for terminal α-2,3-sialyllactosides adds a new source of regulation particular to this galectin (Barake et al., 2020, Hong et al., 2019, Hong et al., 2021). For instance, secreted neuraminidases under inflammatory conditions release sialic acid from cell surface glycans and can switch Gal-8 interactions to other galectins (Barake et al., 2020, Hong et al., 2019, Hong et al., 2021, Nomura et al., 2017). Therefore, N-glycan branching and sialylation/desialylation conditions may impact the extracellular roles of Gal-8, including the mitochondrial adaptations revealed in this study.

## 5 Conclusions

This study highlights the extracellular role of Gal-8 as a crucial regulator of mitochondrial dynamics and cellular metabolism in epithelial cells through mechanisms that involve binding to sialylated glycans and ERK-DRP1 activation. These findings suggest that Gal-8 plays a significant role in cellular adaptation processes, impacting normal physiological and pathological states while opening new avenues for exploring Gal-8 as a potential therapeutic agent to counteract mitochondrial dysfunction and associated diseases.

## Funding sources

This work was supported by Agencia Nacional de Investigación y Desarrollo of Chilean Government [FONDECYT 1181907, 1221796, 1211829 and 1221374, Centro Científico Tecnológico de Excelencia Ciencia y Vida, Fundación Ciencia y Vida, Basal Project FB210008], Vicerrectoría de Investigación y Doctorados de la Universidad San Sebastián – Fondo USS-FIN-25-APCS-15, the scholarship Doctorado Nacional of Agencia Nacional de Investigación y Desarrollo of the Chilean Government [Folio 21221618], and Medical Research Council as part of United Kingdom Research and Innovation [U105170648] and the Wellcome Trust [222503/Z/21/Z].

## CRediT authorship contribution statement

**De la Peña, Adely:** Writing – original draft, Software, Project administration, Methodology, Funding acquisition, Formal analysis, Data curation. **Soza, Andrea:** Writing – original draft, Writing – review & editing, Supervision, Project administration, Funding acquisition, Conceptualization. **Oyanadel, Claudia:** Writing – original draft, Writing – review & editing, Supervision, Conceptualization. **González, Alfonso:** Writing – original draft, Writing – review & editing, Project administration, Funding acquisition, Conceptualization. **Randow, Felix:** Writing – review & editing, Supervision, Project administration, Funding acquisition. **Cancino, Jorge:** Data curation. **Tapia, Diego:** Data curation. **Veloso-Bahamondes, Francisco:** Data curation. **Díaz-Valdivia, Nicole:** Data curation. **Pérez-Molina, Francisca:** Data curation. **Retamal, Claudio:** Software, Methodology, Formal analysis, Data curation.

## Declaration of Competing Interest

The authors declare that they have no known competing financial interests or personal relationships that could have appeared to influence the work reported in this paper.

## Data Availability

Data will be made available on request.

## References

[bib1] Adebayo M., Singh S., Singh A.P., Dasgupta S. (2021). Mitochondrial fusion and fission: the fine-tune balance for cellular homeostasis. FASEB J..

[bib2] Ahmad T., Aggarwal K., Pattnaik B., Mukherjee S., Sethi T., Tiwari B.K., Kumar M., Micheal A., Mabalirajan U., Ghosh B., Sinha Roy S., Agrawal A. (2013). Computational classification of mitochondrial shapes reflects stress and redox state. Cell Death Dis..

[bib3] Albornoz N., Alvarez-Indo J., de la Pena A., Arias-Munoz E., Coca A., Segovia-Miranda F., Kerr B., Budini M., Criollo A., Garcia-Robles M.A., Morselli E., Soza A., Burgos P.V. (2024). Targeting the immunoproteasome in hypothalamic neurons as a novel therapeutic strategy for high-fat diet-induced obesity and metabolic dysregulation. J. Neuroinflamm..

[bib4] Al-Mehdi A.B., Pastukh V.M., Swiger B.M., Reed D.J., Patel M.R., Bardwell G.C., Pastukh V.V., Alexeyev M.F., Gillespie M.N. (2012). Perinuclear mitochondrial clustering creates an oxidant-rich nuclear domain required for hypoxia-induced transcription. Sci. Signal.

[bib5] Anderson C.C., Aivazidis S., Kuzyk C.L., Jain A., Roede J.R. (2018). Acute maneb exposure significantly alters both glycolysis and mitochondrial function in neuroblastoma cells. Toxicol. Sci..

[bib6] Antico Arciuch V.G., Elguero M.E., Poderoso J.J., Carreras M.C. (2012). Mitochondrial regulation of cell cycle and proliferation. Antioxid. Redox Signal.

[bib7] Aparicio-Trejo O.E., Avila-Rojas S.H., Tapia E., Rojas-Morales P., Leon-Contreras J.C., Martinez-Klimova E., Hernandez-Pando R., Sanchez-Lozada L.G., Pedraza-Chaverri J. (2020). Chronic impairment of mitochondrial bioenergetics and beta-oxidation promotes experimental AKI-to-CKD transition induced by folic acid. Free Radic. Biol. Med.

[bib8] Barake F., Soza A., Gonzalez A. (2020). Galectins in the brain: advances in neuroinflammation, neuroprotection and therapeutic opportunities. Curr. Opin. Neurol..

[bib9] Barra J., Crosbourne I., Roberge C.L., Bossardi-Ramos R., Warren J.S.A., Matteson K., Wang L., Jourd'heuil F., Borisov S.M., Bresnahan E., Bravo-Cordero J.J., Dmitriev R.I., Jourd'heuil D., Adam A.P., Lamar J.M., Corr D.T., Barroso M.M. (2024). DMT1-dependent endosome-mitochondria interactions regulate mitochondrial iron translocation and metastatic outgrowth. Oncogene.

[bib10] Boldogh I.R., Pon L.A. (2007). Mitochondria on the move. Trends Cell Biol..

[bib11] Breitzig M.T., Alleyn M.D., Lockey R.F., Kolliputi N. (2018). A mitochondrial delicacy: dynamin-related protein 1 and mitochondrial dynamics. Am. J. Physiol. Cell Physiol..

[bib12] Brewer C.F., Miceli M.C., Baum L.G. (2002). Clusters, bundles, arrays and lattices: novel mechanisms for lectin-saccharide-mediated cellular interactions. Curr. Opin. Struct. Biol..

[bib13] Cagnoni A.J., Troncoso M.F., Rabinovich G.A., Marino K.V., Elola M.T. (2020). Full-length galectin-8 and separate carbohydrate recognition domains: the whole is greater than the sum of its parts?. Biochem Soc. Trans..

[bib14] Caines J.K., Barnes D.A., Berry M.D. (2022). The use of seahorse XF assays to interrogate real-time energy metabolism in cancer cell lines. Methods Mol. Biol..

[bib15] Carcamo C., Pardo E., Oyanadel C., Bravo-Zehnder M., Bull P., Caceres M., Martinez J., Massardo L., Jacobelli S., Gonzalez A., Soza A. (2006). Galectin-8 binds specific beta1 integrins and induces polarized spreading highlighted by asymmetric lamellipodia in Jurkat T cells. Exp. Cell Res.

[bib16] Cerliani J.P., Blidner A.G., Toscano M.A., Croci D.O., Rabinovich G.A. (2017). Translating the 'Sugar Code' into Immune and Vascular Signaling Programs. Trends Biochem Sci..

[bib17] Chakrabarti R., Fung T.S., Kang T., Elonkirjo P.W., Suomalainen A., Usherwood E.J., Higgs H.N. (2022). Mitochondrial dysfunction triggers actin polymerization necessary for rapid glycolytic activation. J. Cell Biol..

[bib18] Chang Y.W., Song Z.H., Chen C.C. (2022). FAK regulates cardiomyocyte mitochondrial fission and function through Drp1. FEBS J..

[bib19] Chang J.Y., Yi H.S., Kim H.W., Shong M. (2017). Dysregulation of mitophagy in carcinogenesis and tumor progression. Biochim Biophys. Acta Bioenerg..

[bib20] Che T.F., Lin C.W., Wu Y.Y., Chen Y.J., Han C.L., Chang Y.L., Wu C.T., Hsiao T.H., Hong T.M., Yang P.C. (2015). Mitochondrial translocation of EGFR regulates mitochondria dynamics and promotes metastasis in NSCLC. Oncotarget.

[bib21] Chen H., Chan D.C. (2017). Mitochondrial dynamics in regulating the unique phenotypes of cancer and stem cells. Cell Metab..

[bib22] Chen W., Zhao H., Li Y. (2023). Mitochondrial dynamics in health and disease: mechanisms and potential targets. Signal Transduct. Target Ther..

[bib23] Chiu Y.H., Lin S.A., Kuo C.H., Li C.J. (2021). Molecular machinery and pathophysiology of mitochondrial dynamics. Front Cell Dev. Biol..

[bib24] Clague M.J., Urbe S. (2025). Diverse routes to mitophagy governed by ubiquitylation and mitochondrial import. Trends Cell Biol..

[bib25] Collins T.J., Berridge M.J., Lipp P., Bootman M.D. (2002). Mitochondria are morphologically and functionally heterogeneous within cells. EMBO J..

[bib26] Daga P., Thurakkal B., Rawal S., Das T. (2024). Matrix stiffening promotes perinuclear clustering of mitochondria. Mol. Biol. Cell.

[bib27] Deepak K., Roy P.K., Das C.K., Mukherjee B., Mandal M. (2024). Mitophagy at the crossroads of cancer development: exploring the role of mitophagy in tumor progression and therapy resistance. Biochim Biophys. Acta Mol. Cell Res.

[bib28] Deng Y., Jia F., Jiang P., Chen L., Xing L., Shen X., Li L., Huang Y. (2023). Biomimetic nanoparticle synchronizing pyroptosis induction and mitophagy inhibition for anti-tumor therapy. Biomaterials.

[bib29] Dennis J.W., Lau K.S., Demetriou M., Nabi I.R. (2009). Adaptive regulation at the cell surface by N-glycosylation. Traffic.

[bib30] Desai R., East D.A., Hardy L., Faccenda D., Rigon M., Crosby J., Alvarez M.S., Singh A., Mainenti M., Hussey L.K., Bentham R., Szabadkai G., Zappulli V., Dhoot G.K., Romano L.E., Xia D., Coppens I., Hamacher-Brady A., Chapple J.P., Campanella M. (2020). Mitochondria form contact sites with the nucleus to couple prosurvival retrograde response. Sci. Adv..

[bib31] Diskin S., Chen W.S., Cao Z., Gyawali S., Gong H., Soza A., Gonzalez A., Panjwani N. (2012). Galectin-8 promotes cytoskeletal rearrangement in trabecular meshwork cells through activation of Rho signaling. PLoS One.

[bib32] Divakaruni A.S., Brand M.D. (2011). The regulation and physiology of mitochondrial proton leak. Physiol. (Bethesda).

[bib33] Dong Y., Zhang X. (2024). Targeting cellular mitophagy as a strategy for human cancers. Front Cell Dev. Biol..

[bib34] Drake J.C., Yan Z. (2017). Mitophagy in maintaining skeletal muscle mitochondrial proteostasis and metabolic health with ageing. J. Physiol..

[bib35] Eberhardt E.L., Ludlam A.V., Tan Z., Cianfrocco M.A. (2020). Miro: A molecular switch at the center of mitochondrial regulation. Protein Sci..

[bib36] Elola M.T., Ferragut F., Cardenas Delgado V.M., Nugnes L.G., Gentilini L., Laderach D., Troncoso M.F., Compagno D., Wolfenstein-Todel C., Rabinovich G.A. (2014). Expression, localization and function of galectin-8, a tandem-repeat lectin, in human tumors. Histol. Histopathol..

[bib37] Flannery P.J., Trushina E. (2019). Mitochondrial dynamics and transport in Alzheimer's disease. Mol. Cell Neurosci..

[bib38] Friedman J.R., Nunnari J. (2014). Mitochondrial form and function. Nature.

[bib39] Furnish M., Caino M.C. (2020). Altered mitochondrial trafficking as a novel mechanism of cancer metastasis. Cancer Rep. (Hoboken).

[bib40] Gan X., Huang S., Wu L., Wang Y., Hu G., Li G., Zhang H., Yu H., Swerdlow R.H., Chen J.X., Yan S.S. (2014). Inhibition of ERK-DLP1 signaling and mitochondrial division alleviates mitochondrial dysfunction in Alzheimer's disease cybrid cell. Biochim Biophys. Acta.

[bib41] Gomes L.C., Di Benedetto G., Scorrano L. (2011). During autophagy mitochondria elongate, are spared from degradation and sustain cell viability. Nat. Cell Biol..

[bib42] Hara Y., Yuk F., Puri R., Janssen W.G., Rapp P.R., Morrison J.H. (2014). Presynaptic mitochondrial morphology in monkey prefrontal cortex correlates with working memory and is improved with estrogen treatment. Proc. Natl. Acad. Sci. USA.

[bib43] Harper J.W., Ordureau A., Heo J.M. (2018). Building and decoding ubiquitin chains for mitophagy. Nat. Rev. Mol. Cell Biol..

[bib44] Hirabayashi J., Hashidate T., Arata Y., Nishi N., Nakamura T., Hirashima M., Urashima T., Oka T., Futai M., Muller W.E., Yagi F., Kasai K. (2002). Oligosaccharide specificity of galectins: a search by frontal affinity chromatography. Biochim Biophys. Acta.

[bib45] Hong M.H., Lin W.H., Weng I.C., Hung Y.H., Chen H.L., Chen H.Y., Chen P., Lin C.H., Yang W.Y., Liu F.T. (2019). Intracellular galectins control cellular responses commensurate with cell surface carbohydrate composition. Glycobiology.

[bib46] Hong M.H., Weng I.C., Li F.Y., Lin W.H., Liu F.T. (2021). Intracellular galectins sense cytosolically exposed glycans as danger and mediate cellular responses. J. Biomed. Sci..

[bib47] Hoyer M.J., Swarup S., Harper J.W. (2022). Mechanisms controlling selective elimination of damaged lysosomes. Curr. Opin. Physiol..

[bib48] Huang D., Chen S., Xiong D., Wang H., Zhu L., Wei Y., Li Y., Zou S. (2023). Mitochondrial dynamics: working with the cytoskeleton and intracellular organelles to mediate mechanotransduction. Aging Dis..

[bib49] Ihenacho U.K., Meacham K.A., Harwig M.C., Widlansky M.E., Hill R.B. (2021). Mitochondrial fission protein 1: emerging roles in organellar form and function in health and disease. Front Endocrinol. (Lausanne).

[bib50] Jastroch M., Divakaruni A.S., Mookerjee S., Treberg J.R., Brand M.D. (2010). Mitochondrial proton and electron leaks. Essays Biochem.

[bib51] Jenkins B.C., Neikirk K., Katti P., Claypool S.M., Kirabo A., McReynolds M.R., Hinton A. (2024). Mitochondria in disease: changes in shapes and dynamics. Trends Biochem Sci..

[bib52] Jheng H.F., Tsai P.J., Guo S.M., Kuo L.H., Chang C.S., Su I.J., Chang C.R., Tsai Y.S. (2012). Mitochondrial fission contributes to mitochondrial dysfunction and insulin resistance in skeletal muscle. Mol. Cell Biol..

[bib53] Jia J., Abudu Y.P., Claude-Taupin A., Gu Y., Kumar S., Choi S.W., Peters R., Mudd M.H., Allers L., Salemi M., Phinney B., Johansen T., Deretic V. (2019). Galectins control MTOR and AMPK in response to lysosomal damage to induce autophagy. Autophagy.

[bib54] Jia J., Claude-Taupin A., Gu Y., Choi S.W., Peters R., Bissa B., Mudd M.H., Allers L., Pallikkuth S., Lidke K.A., Salemi M., Phinney B., Mari M., Reggiori F., Deretic V. (2020). Galectin-3 coordinates a cellular system for lysosomal repair and removal. Dev. Cell.

[bib55] Jiang F., Jia Y., Cohen I. (2002). Fibronectin- and protein kinase C-mediated activation of ERK/MAPK are essential for proplateletlike formation. Blood.

[bib56] Johannes L., Jacob R., Leffler H. (2018). Galectins at a glance. J. Cell Sci..

[bib57] Ko A.R., Hyun H.W., Min S.J., Kim J.E. (2016). The Differential DRP1 phosphorylation and mitochondrial dynamics in the regional specific astroglial death induced by status epilepticus. Front Cell Neurosci..

[bib58] Koncha R.R., Ramachandran G., Sepuri N.B.V., Ramaiah K.V.A. (2021). CCCP-induced mitochondrial dysfunction - characterization and analysis of integrated stress response to cellular signaling and homeostasis. FEBS J..

[bib59] Kwon D., Park E., Sesaki H., Kang S.J. (2017). Carbonyl cyanide 3-chlorophenylhydrazone (CCCP) suppresses STING-mediated DNA sensing pathway through inducing mitochondrial fission. Biochem Biophys. Res Commun..

[bib60] Lacombe A., Scorrano L. (2024). The interplay between mitochondrial dynamics and autophagy: From a key homeostatic mechanism to a driver of pathology. Semin Cell Dev. Biol..

[bib61] Lampert M.A., Orogo A.M., Najor R.H., Hammerling B.C., Leon L.J., Wang B.J., Kim T., Sussman M.A., Gustafsson A.B. (2019). BNIP3L/NIX and FUNDC1-mediated mitophagy is required for mitochondrial network remodeling during cardiac progenitor cell differentiation. Autophagy.

[bib62] Lawrence E.J., Boucher E., Mandato C.A. (2016). Mitochondria-cytoskeleton associations in mammalian cytokinesis. Cell Div..

[bib63] Ledesma A., de Lacoba M.G., Rial E. (2002). The mitochondrial uncoupling proteins. Genome Biol..

[bib64] Lee S., Son J.Y., Lee J., Cheong H. (2023). Unraveling the intricacies of autophagy and mitophagy: implications in cancer biology. Cells.

[bib65] Li Z., Munim M.B., Sharygin D.A., Bevis B.J., Vander Heiden M.G. (2024). Understanding the Warburg effect in cancer. Cold Spring Harb. Perspect. Med.

[bib66] Li W., Sancho A., Chung W.L., Vinik Y., Groll J., Zick Y., Medalia O., Bershadsky A.D., Geiger B. (2021). Differential cellular responses to adhesive interactions with galectin-8- and fibronectin-coated substrates. J. Cell Sci..

[bib67] Liesa M., Shirihai O.S. (2013). Mitochondrial dynamics in the regulation of nutrient utilization and energy expenditure. Cell Metab..

[bib68] Lionetti L., Mollica M.P., Donizzetti I., Gifuni G., Sica R., Pignalosa A., Cavaliere G., Gaita M., De Filippo C., Zorzano A., Putti R. (2014). High-lard and high-fish-oil diets differ in their effects on function and dynamic behaviour of rat hepatic mitochondria. PLoS One.

[bib69] Liu X., Hajnoczky G. (2011). Altered fusion dynamics underlie unique morphological changes in mitochondria during hypoxia-reoxygenation stress. Cell Death Differ..

[bib70] Liu Y.J., McIntyre R.L., Janssens G.E., Houtkooper R.H. (2020). Mitochondrial fission and fusion: A dynamic role in aging and potential target for age-related disease. Mech. Ageing Dev..

[bib71] Long Q., Zhao D., Fan W., Yang L., Zhou Y., Qi J., Wang X., Liu X. (2015). Modeling of Mitochondrial Donut Formation. Biophys. J..

[bib72] Lopez-Mejia I.C., Fajas L. (2015). Cell cycle regulation of mitochondrial function. Curr. Opin. Cell Biol..

[bib73] Lovas J.R., Wang X. (2013). The meaning of mitochondrial movement to a neuron's life. Biochim Biophys. Acta.

[bib74] Lu X., Chen L., Chen Y., Shao Q., Qin W. (2015). Bafilomycin A1 inhibits the growth and metastatic potential of the BEL-7402 liver cancer and HO-8910 ovarian cancer cell lines and induces alterations in their microRNA expression. Exp. Ther. Med.

[bib75] Martinez-Reyes I., Chandel N.S. (2021). Cancer metabolism: looking forward. Nat. Rev. Cancer.

[bib76] Mauro-Lizcano M., Sotgia F., Lisanti M.P. (2024). Mitophagy and cancer: role of BNIP3/BNIP3L as energetic drivers of stemness features, ATP production, proliferation, and cell migration. Aging (Albany NY).

[bib77] Mauvezin C., Neufeld T.P. (2015). Bafilomycin A1 disrupts autophagic flux by inhibiting both V-ATPase-dependent acidification and Ca-P60A/SERCA-dependent autophagosome-lysosome fusion. Autophagy.

[bib78] Meacham C.E., DeVilbiss A.W., Morrison S.J. (2022). Metabolic regulation of somatic stem cells in vivo. Nat. Rev. Mol. Cell Biol..

[bib79] Miao M.Q., Han Y.B., Liu L. (2023). Mitophagy in metabolic syndrome. J. Clin. Hypertens. (Greenwich).

[bib80] Mitra K., Wunder C., Roysam B., Lin G., Lippincott-Schwartz J. (2009). A hyperfused mitochondrial state achieved at G1-S regulates cyclin E buildup and entry into S phase. Proc. Natl. Acad. Sci. USA.

[bib81] Mookerjee S.A., Goncalves R.L.S., Gerencser A.A., Nicholls D.G., Brand M.D. (2015). The contributions of respiration and glycolysis to extracellular acid production. Biochim Biophys. Acta.

[bib82] Moore A.S., Holzbaur E.L.F. (2018). Mitochondrial-cytoskeletal interactions: dynamic associations that facilitate network function and remodeling. Curr. Opin. Physiol..

[bib83] Mori M.P., Penjweini R., Ma J., Alspaugh G., Andreoni A., Kim Y.C., Wang P.Y., Knutson J.R., Hwang P.M. (2023). Mitochondrial respiration reduces exposure of the nucleus to oxygen. J. Biol. Chem..

[bib84] Nabi I.R., Shankar J., Dennis J.W. (2015). The galectin lattice at a glance. J. Cell Sci..

[bib85] Naik P.P., Praharaj P.P., Bhol C.S., Panigrahi D.P., Mahapatra K.K., Patra S., Saha S., Bhutia S.K. (2019). Mitochondrial heterogeneity in stem cells. Adv. Exp. Med Biol..

[bib86] Nomura K., Vilalta A., Allendorf D.H., Hornik T.C., Brown G.C. (2017). Activated microglia desialylate and phagocytose cells via neuraminidase, galectin-3, and mer tyrosine kinase. J. Immunol..

[bib87] Oyanadel C., Holmes C., Pardo E., Retamal C., Shaughnessy R., Smith P., Cortes P., Bravo-Zehnder M., Metz C., Feuerhake T., Romero D. t, Roa J.C., Montecinos V., Soza A., Gonzalez A. (2018). Galectin-8 induces partial epithelial-mesenchymal transition with invasive tumorigenic capabilities involving a FAK/EGFR/proteasome pathway in Madin-Darby canine kidney cells. Mol. Biol. Cell.

[bib88] Pagliuso A., Cossart P., Stavru F. (2018). The ever-growing complexity of the mitochondrial fission machinery. Cell Mol. Life Sci..

[bib89] Palikaras K., Lionaki E., Tavernarakis N. (2015). Balancing mitochondrial biogenesis and mitophagy to maintain energy metabolism homeostasis. Cell Death Differ..

[bib90] Palikaras K., Lionaki E., Tavernarakis N. (2015). Coupling mitogenesis and mitophagy for longevity. Autophagy.

[bib91] Palikaras K., Lionaki E., Tavernarakis N. (2016). Mitophagy: in sickness and in health. Mol. Cell Oncol..

[bib92] Palikaras K., Lionaki E., Tavernarakis N. (2018). Mechanisms of mitophagy in cellular homeostasis, physiology and pathology. Nat. Cell Biol..

[bib93] Palikaras K., Tavernarakis N. (2014). Mitochondrial homeostasis: the interplay between mitophagy and mitochondrial biogenesis. Exp. Gerontol..

[bib94] Park M.K., Ashby M.C., Erdemli G., Petersen O.H., Tepikin A.V. (2001). Perinuclear, perigranular and sub-plasmalemmal mitochondria have distinct functions in the regulation of cellular calcium transport. EMBO J..

[bib95] Patnaik S.K., Stanley P. (2006). Lectin-resistant CHO glycosylation mutants. Methods Enzym..

[bib96] Perez-Moreno E., Oyanadel C., de la Pena A., Hernandez R., Perez-Molina F., Metz C., Gonzalez A., Soza A. (2024). Galectins in epithelial-mesenchymal transition: roles and mechanisms contributing to tissue repair, fibrosis and cancer metastasis. Biol. Res.

[bib97] Perez-Moreno E., Toledo T., Campusano P., Zuniga S., Azocar L., Feuerhake T., Mendez G.P., Labarca M., Perez-Molina F., de la Pena A., Herrera-Cid C., Ehrenfeld P., Godoy A.S., Gonzalez A., Soza A. (2024). Galectin-8 counteracts folic acid-induced acute kidney injury and prevents its transition to fibrosis. Biomed. Pharm..

[bib98] Perry S.W., Norman J.P., Barbieri J., Brown E.B., Gelbard H.A. (2011). Mitochondrial membrane potential probes and the proton gradient: a practical usage guide. Biotechniques.

[bib99] Picard M., Shirihai O.S. (2022). Mitochondrial signal transduction. Cell Metab..

[bib100] Potter M., Newport E., Morten K.J. (2016). The Warburg effect: 80 years on. Biochem Soc. Trans..

[bib101] Prieto J., Leon M., Ponsoda X., Sendra R., Bort R., Ferrer-Lorente R., Raya A., Lopez-Garcia C., Torres J. (2016). Early ERK1/2 activation promotes DRP1-dependent mitochondrial fission necessary for cell reprogramming. Nat. Commun..

[bib102] Pyakurel A., Savoia C., Hess D., Scorrano L. (2015). Extracellular regulated kinase phosphorylates mitofusin 1 to control mitochondrial morphology and apoptosis. Mol. Cell.

[bib103] Rabinovich G.A., van Kooyk Y., Cobb B.A. (2012). Glycobiology of immune responses. Ann. N. Y Acad. Sci..

[bib104] Ravenhill B.J., Boyle K.B., von Muhlinen N., Ellison C.J., Masson G.R., Otten E.G., Foeglein A., Williams R., Randow F. (2019). The Cargo Receptor NDP52 Initiates Selective Autophagy by Recruiting the ULK Complex to Cytosol-Invading Bacteria. Mol. Cell.

[bib105] Rohn J.L., Patel J.V., Neumann B., Bulkescher J., McHedlishvili N., McMullan R.C., Quintero O.A., Ellenberg J., Baum B. (2014). Myo19 ensures symmetric partitioning of mitochondria and coupling of mitochondrial segregation to cell division. Curr. Biol..

[bib106] Rolland S.G., Motori E., Memar N., Hench J., Frank S., Winklhofer K.F., Conradt B. (2013). Impaired complex IV activity in response to loss of LRPPRC function can be compensated by mitochondrial hyperfusion. Proc. Natl. Acad. Sci. USA.

[bib107] Sessions D.T., Kashatus D.F. (2021). Mitochondrial dynamics in cancer stem cells. Cell Mol. Life Sci..

[bib108] Shatz-Azoulay H., Vinik Y., Isaac R., Kohler U., Lev S., Zick Y. (2020). The Animal Lectin Galectin-8 Promotes Cytokine Expression and Metastatic Tumor Growth in Mice. Sci. Rep..

[bib109] Smith P.C., Metz C., de la Pena A., Oyanadel C., Avila P., Arancibia R., Vicuna L., Retamal C., Barake F., Gonzalez A., Soza A. (2020). Galectin-8 mediates fibrogenesis induced by cyclosporine in human gingival fibroblasts. J. Periodontal Res.

[bib110] Stanley P. (1989). Chinese hamster ovary cell mutants with multiple glycosylation defects for production of glycoproteins with minimal carbohydrate heterogeneity. Mol. Cell Biol..

[bib111] Suh J., Kim N.K., Shim W., Lee S.H., Kim H.J., Moon E., Sesaki H., Jang J.H., Kim J.E., Lee Y.S. (2023). Mitochondrial fragmentation and donut formation enhance mitochondrial secretion to promote osteogenesis. Cell Metab..

[bib112] Sun N., Malide D., Liu J., Rovira I.I., Combs C.A., Finkel T. (2017). A fluorescence-based imaging method to measure in vitro and in vivo mitophagy using mt-Keima. Nat. Protoc..

[bib113] Suomalainen A., Nunnari J. (2024). Mitochondria at the crossroads of health and disease. Cell.

[bib114] Tabara L.C., Morris J.L., Prudent J. (2021). The complex dance of organelles during mitochondrial division. Trends Cell Biol..

[bib115] Tang S., Geng Y., Lin Q. (2024). The role of mitophagy in metabolic diseases and its exercise intervention. Front Physiol..

[bib116] Tang J., Peng W., Ji J., Peng C., Wang T., Yang P., Gu J., Feng Y., Jin K., Wang X., Sun Y. (2023). GPR176 Promotes Cancer Progression by Interacting with G Protein GNAS to Restrain Cell Mitophagy in Colorectal Cancer. Adv. Sci. (Weinh. ).

[bib117] Thurston T.L., Boyle K.B., Allen M., Ravenhill B.J., Karpiyevich M., Bloor S., Kaul A., Noad J., Foeglein A., Matthews S.A., Komander D., Bycroft M., Randow F. (2016). Recruitment of TBK1 to cytosol-invading Salmonella induces WIPI2-dependent antibacterial autophagy. EMBO J..

[bib118] Thurston T.L., Wandel M.P., von Muhlinen N., Foeglein A., Randow F. (2012). Galectin 8 targets damaged vesicles for autophagy to defend cells against bacterial invasion. Nature.

[bib119] Tilokani L., Nagashima S., Paupe V., Prudent J. (2018). Mitochondrial dynamics: overview of molecular mechanisms. Essays Biochem.

[bib120] Troncoso M.F., Elola M.T., Blidner A.G., Sarrias L., Espelt M.V., Rabinovich G.A. (2023). The universe of galectin-binding partners and their functions in health and disease. J. Biol. Chem..

[bib121] Vander Heiden M.G., DeBerardinis R.J. (2017). Understanding the Intersections between Metabolism and Cancer Biology. Cell.

[bib122] Vicuna L., Pardo E., Curkovic C., Doger R., Oyanadel C., Metz C., Massardo L., Gonzalez A., Soza A. (2013). Galectin-8 binds to LFA-1, blocks its interaction with ICAM-1 and is counteracted by anti-Gal-8 autoantibodies isolated from lupus patients. Biol. Res.

[bib123] Vyas S., Zaganjor E., Haigis M.C. (2016). Mitochondria and Cancer. Cell.

[bib124] Wakabayashi J., Zhang Z., Wakabayashi N., Tamura Y., Fukaya M., Kensler T.W., Iijima M., Sesaki H. (2009). The dynamin-related GTPase Drp1 is required for embryonic and brain development in mice. J. Cell Biol..

[bib125] Wang S., Long H., Hou L., Feng B., Ma Z., Wu Y., Zeng Y., Cai J., Zhang D.W., Zhao G. (2023). The mitophagy pathway and its implications in human diseases. Signal Transduct. Target Ther..

[bib126] Wang S., Tan J., Miao Y., Zhang Q. (2022). Mitochondrial dynamics, mitophagy, and mitochondria-endoplasmic reticulum contact sites crosstalk under hypoxia. Front Cell Dev. Biol..

[bib127] Westermann B. (2012). Bioenergetic role of mitochondrial fusion and fission. Biochim Biophys. Acta.

[bib128] Wolkow C.A., Iser W.B. (2006). Uncoupling protein homologs may provide a link between mitochondria, metabolism and lifespan. Ageing Res Rev..

[bib129] Xie Z., Xie Y., Xu Y., Zhou H., Xu W., Dong Q. (2014). Bafilomycin A1 inhibits autophagy and induces apoptosis in MG63 osteosarcoma cells. Mol. Med Rep..

[bib130] Xue D., Zhou X., Qiu J. (2020). Emerging role of NRF2 in ROS-mediated tumor chemoresistance. Biomed. Pharm..

[bib131] Yan Y., Jiang K., Liu P., Zhang X., Dong X., Gao J., Liu Q., Barr M.P., Zhang Q., Hou X., Meng S., Gong P. (2016). Bafilomycin A1 induces caspase-independent cell death in hepatocellular carcinoma cells via targeting of autophagy and MAPK pathways. Sci. Rep..

[bib132] Yoo I., Ahn I., Lee J., Lee N. (2024). Extracellular flux assay (Seahorse assay): Diverse applications in metabolic research across biological disciplines. Mol. Cells.

[bib133] Yu T., Jhun B.S., Yoon Y. (2011). High-glucose stimulation increases reactive oxygen species production through the calcium and mitogen-activated protein kinase-mediated activation of mitochondrial fission. Antioxid. Redox Signal.

[bib134] Zeng F., Zhang S., Hao Z., Duan S., Meng Y., Li P., Dong J., Lin Y. (2018). Efficient strategy for introducing large and multiple changes in plasmid DNA. Sci. Rep..

[bib135] Zick Y. (2022). Galectin-8, cytokines, and the storm. Biochem Soc. Trans..

