# Supplementary material for: Galectin-8 drives ERK-dependent mitochondrial fragmentation, perinuclear relocation and mitophagy, with metabolic adaptations for cell proliferation
Source: Eur J Cell Biol. 2025 Jun;104(2):151488. doi: 10.1016/j.ejcb.2025.151488 (PMC12162348; doi:10.1016/j.ejcb.2025.151488)
Supplement: Supplementary file 1 — Supplementary material [file mmc1.docx]

**Supplementary Figure 1. Recombinant protein purification.**

The purification process of Gal-8. The pETM30 vector was used for the expression of recombinant proteins, fused to His-GST in E. coli strain BL21. The lysates of bacteria were added to a Glutathione-Superflow Resin column, and then incubated with TEV protease. The supernatant was incubated with resin Ni-NTA magnetic agarose beads to remove the TEV protease and the GST-Sepharose. The resulting purification fractions were analyzed by SDS-PAGE and stained with Coomassie.

**Supplementary Figure 2. Gal-8 induces proliferation accompanied by fragmentation and perinuclear distribution of mitochondria in CHO-K1 and RPTEC cells.**

A) 3D Confocal microscopy images showing MitoTracker CMTMRos stained mitochondria. Huygens Software rendered images of mitochondria of CHO-K1 and RPTEC cells that were treated with Gal-8 (50 μg/ml) for 24 hours. Graphs showing the length and surface of mitochondria in B) CHO-K1 and C) RPTEC cells. D) Graphs showing the number of mitochondria from CHO-K1 and RPTEC cells. E) Percentage of mitochondria within 3 μm of the nucleus in CHO-K1 and RPTEC cells. F) Ki67 positive cell rate in RPTEC cells. (Mean ± s.d., n=3, 15 cells per experiment, One-Way ANOVA with a posterior Tukey, *p<0.05, **p<0.01, ***p<0.001). Scale bar = 10μm for A) and 100μm for F).

**Supplementary Figure 3. Gal-8 induces fragmentation and perinuclear distribution of mitochondria in a carbohydrate recognition-dependent manner in CHO-K1 cells.**

Confocal 3D microscopy images showing MitoTracker CMTMRos stained mitochondria and Hoechst-stained nuclei in fixed CHO-K1 cells treated with Gal-8, Gal-8-R69H, Gal-8-R275H (50 µg/ml) for 24 hours A) Huygens Software rendered 3D surface reconstructions of mitochondria in CHO-K1 cells. B) Graphs showing the length and surface of mitochondria. C) Percentage of mitochondria within 3 μm of the nucleus. (Mean ± s.d., n=3, 15 cells per experiment, One-Way ANOVA with a posterior Tukey, *p<0.05, **p<0.01, ***p<0.001). Scale bar = 10μm.

**Supplementary Figure 4. Gal-8-induced fragmentation and redistribution of mitochondria to the perinuclear zone depend on ERK-mediated DRP1-increased expression in RPTEC cells.**

Z-Stack Confocal microscopy images showing MitoTracker CMTMRos stained mitochondria and Hoechst-stained nuclei in fixed RPTEC cells that were treated with Gal-8 (50 μg/ml) in the presence or absence of MEK inhibitor PD98059 (25 μM) for 24 hours. A) Huygens Software rendered 3D surface reconstructions of mitochondria in RPTEC cells. B) Length and surface of mitochondria. C) Percentage of mitochondria within 3 μm of the nucleus. D) Immunoblots of DRP1, pERK, and ERK of RPTEC cells treated as indicated. The graphs show the quantification of D). (Mean ± s.d. n=3, 15 cells per experiment, One-Way ANOVA with a posterior Tukey, *p<0.05, ***p<0.001). Scale bar = 10μm.

**Supplementary Figure 5. Gal-8 induces actin filament rearrangement.**MDCK-MT-GFP cells were treated with either vehicle or Gal-8 (50 μg/ml) for 24 hours, with or without Cytochalasin D (2 μM). Actin filaments were stained using phalloidin. Three independent experiments were performed. Scale bar = 10μm.

**Supplementary Figure 6.** **Fibronectin induces actin filament rearrangement but does not cause mitochondrial fragmentation or perinuclear redistribution.**

MDCK-MT-GFP cells were seeded in glass-bottom Petri dishes coated with either 50 μg/ml of Gal-8, 25 μg/ml of fibronectin, or a combination of 50 μg/ml Gal-8 and 25 μg/ml fibronectin, and incubated for 24 hours. As a control, the dishes were coated with PBS. Three independent experiments were performed. Scale bar = 10μm.
