# Supplementary figures and images for: Galectin-8 drives ERK-dependent mitochondrial fragmentation, perinuclear relocation and mitophagy, with metabolic adaptations for cell proliferation
Source: Eur J Cell Biol. 2025 Jun;104(2):151488. doi: 10.1016/j.ejcb.2025.151488 (PMC12162348; doi:10.1016/j.ejcb.2025.151488)

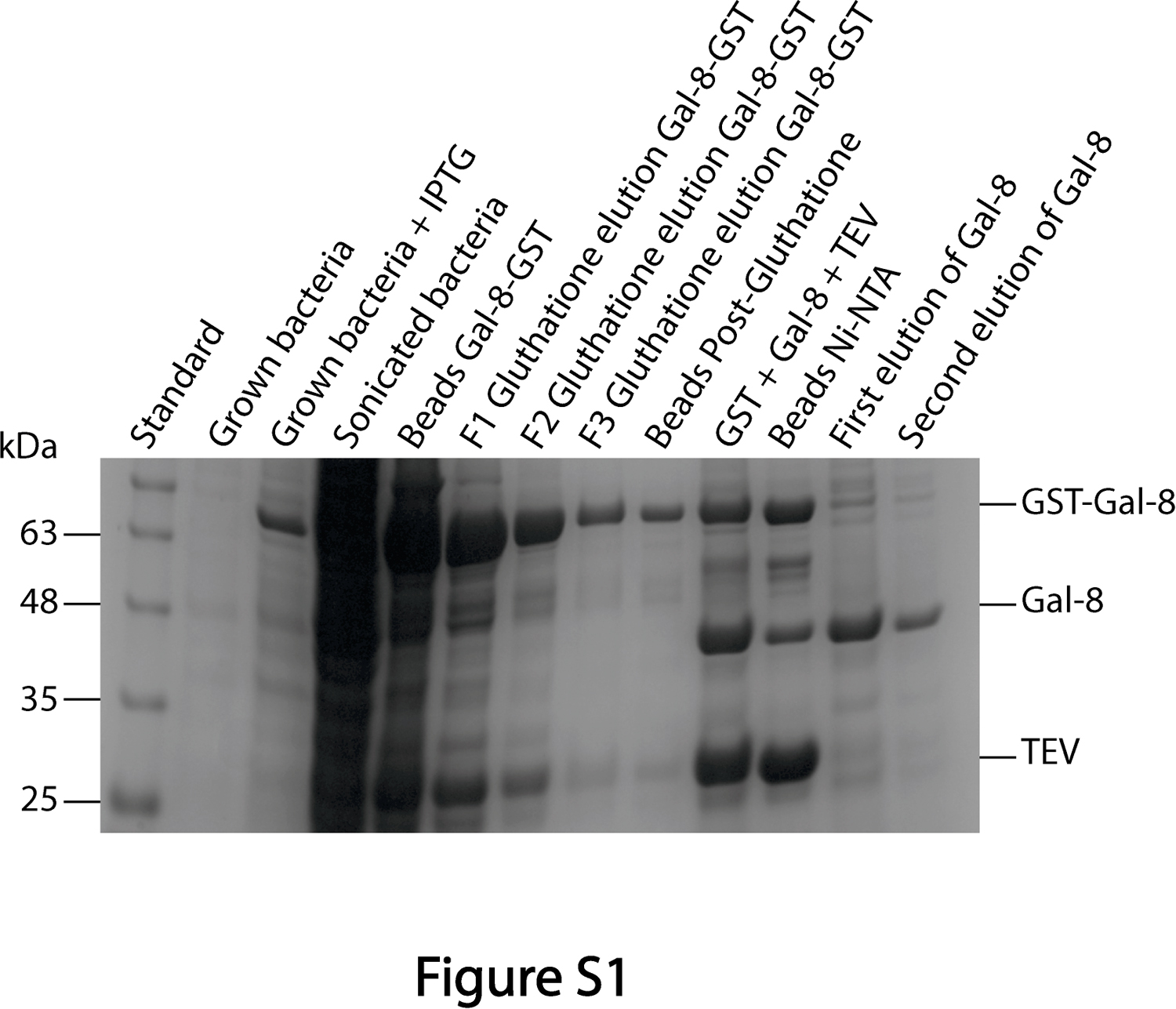

Supplement: Supplementary file 2 — Supplementary material [file mmc2.jpg]

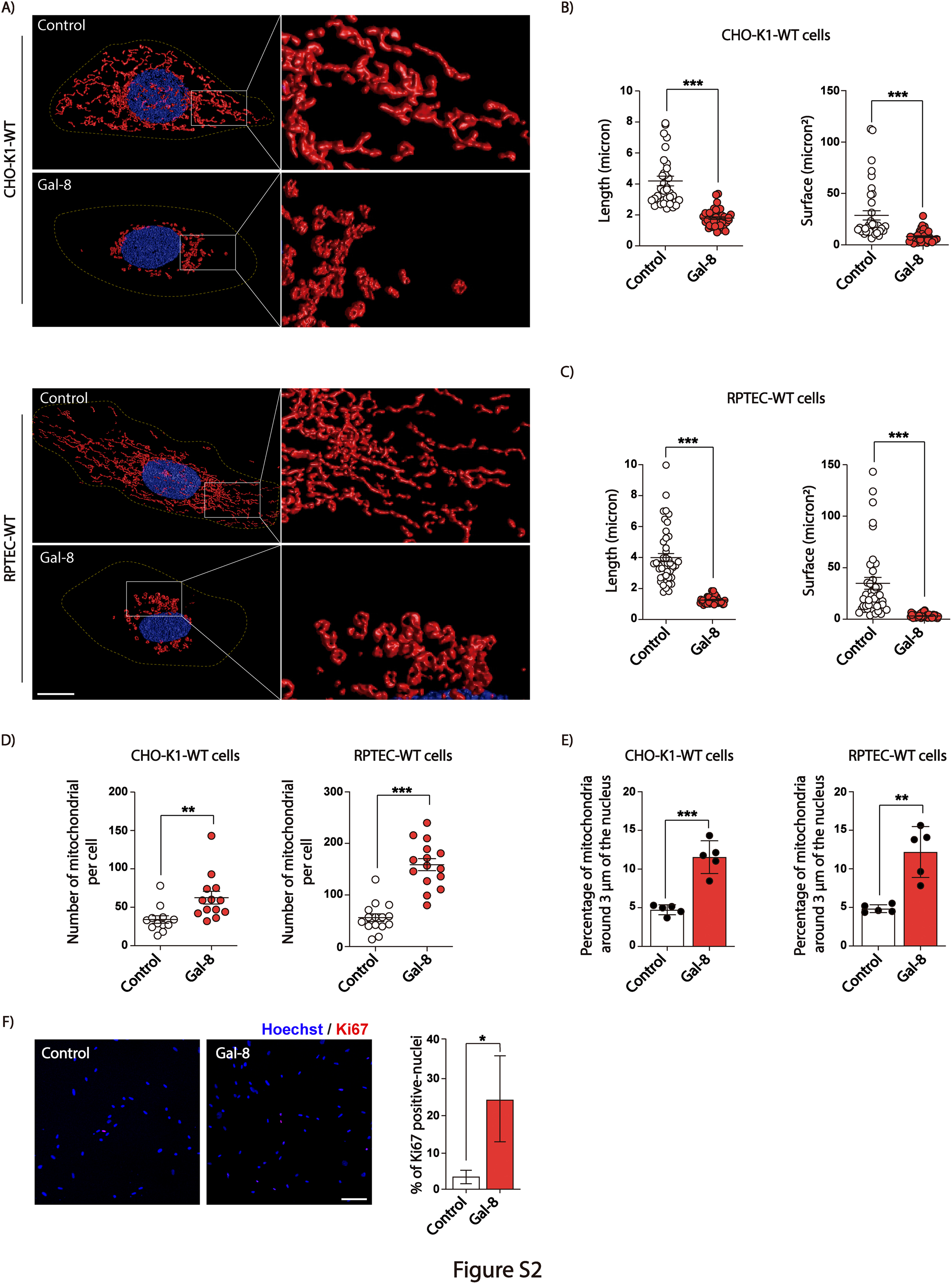

Supplement: Supplementary file 3 — Supplementary material [file mmc3.jpg]

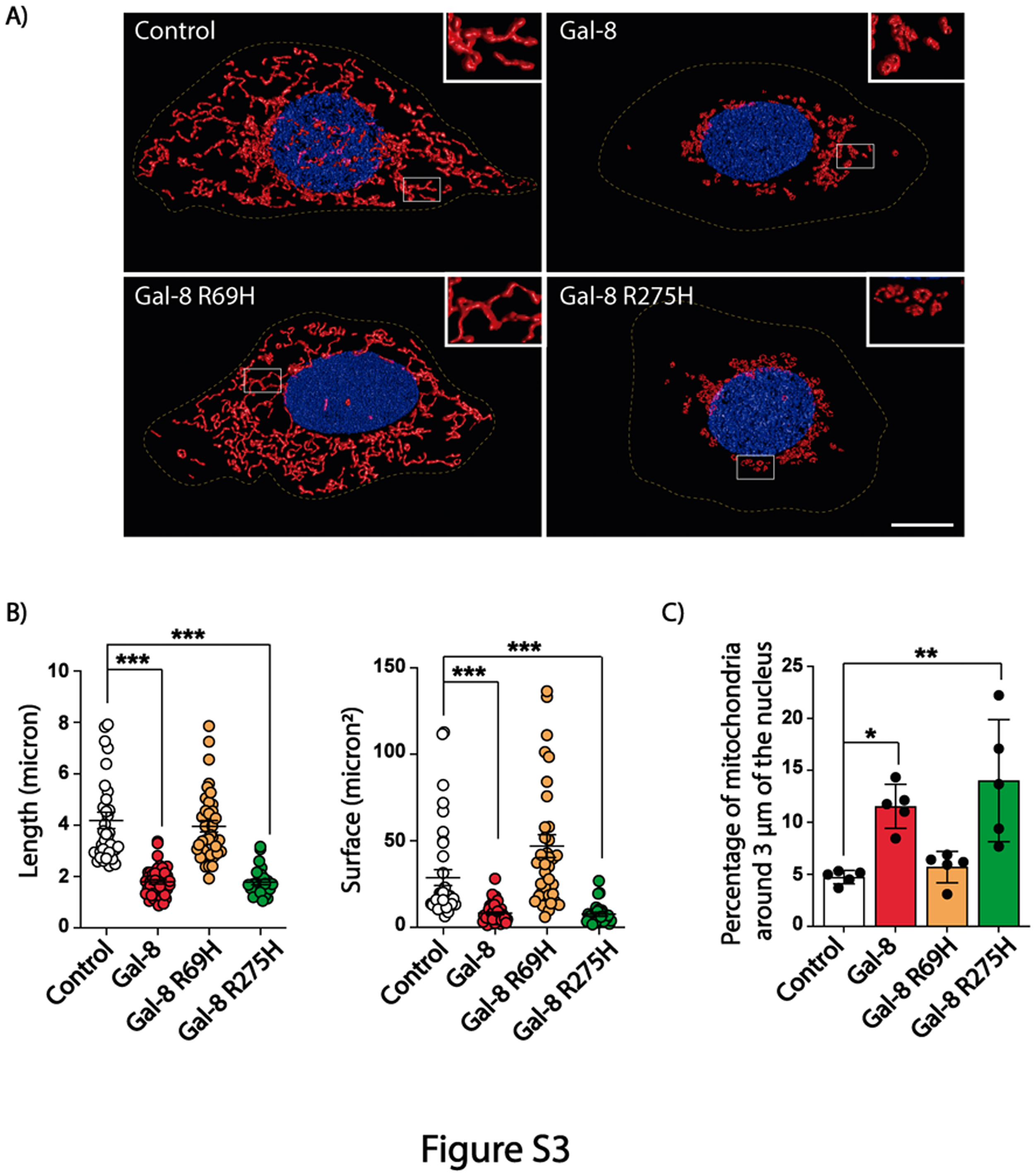

Supplement: Supplementary file 4 — Supplementary material [file mmc4.jpg]

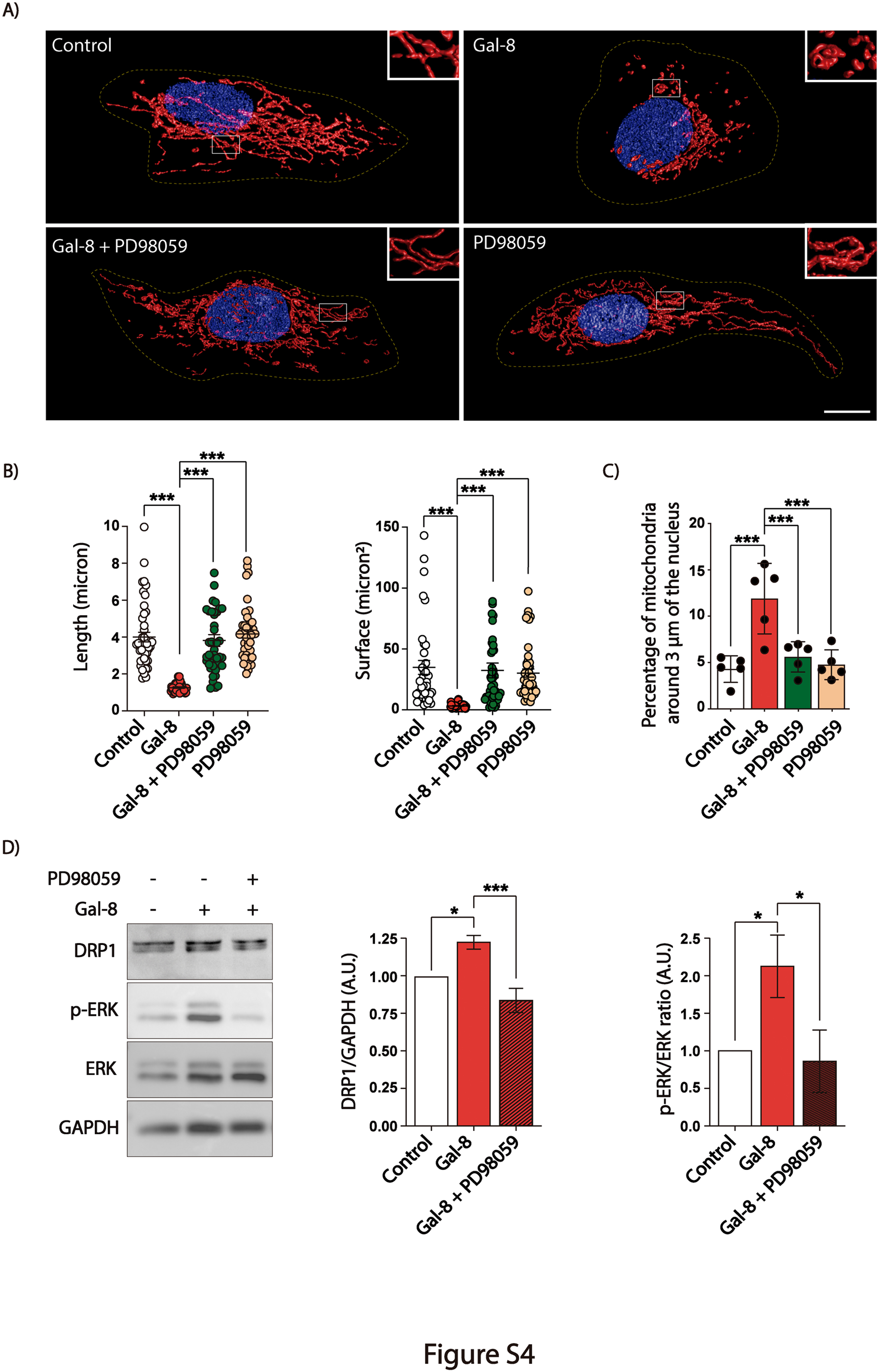

Supplement: Supplementary file 5 — Supplementary material [file mmc5.jpg]

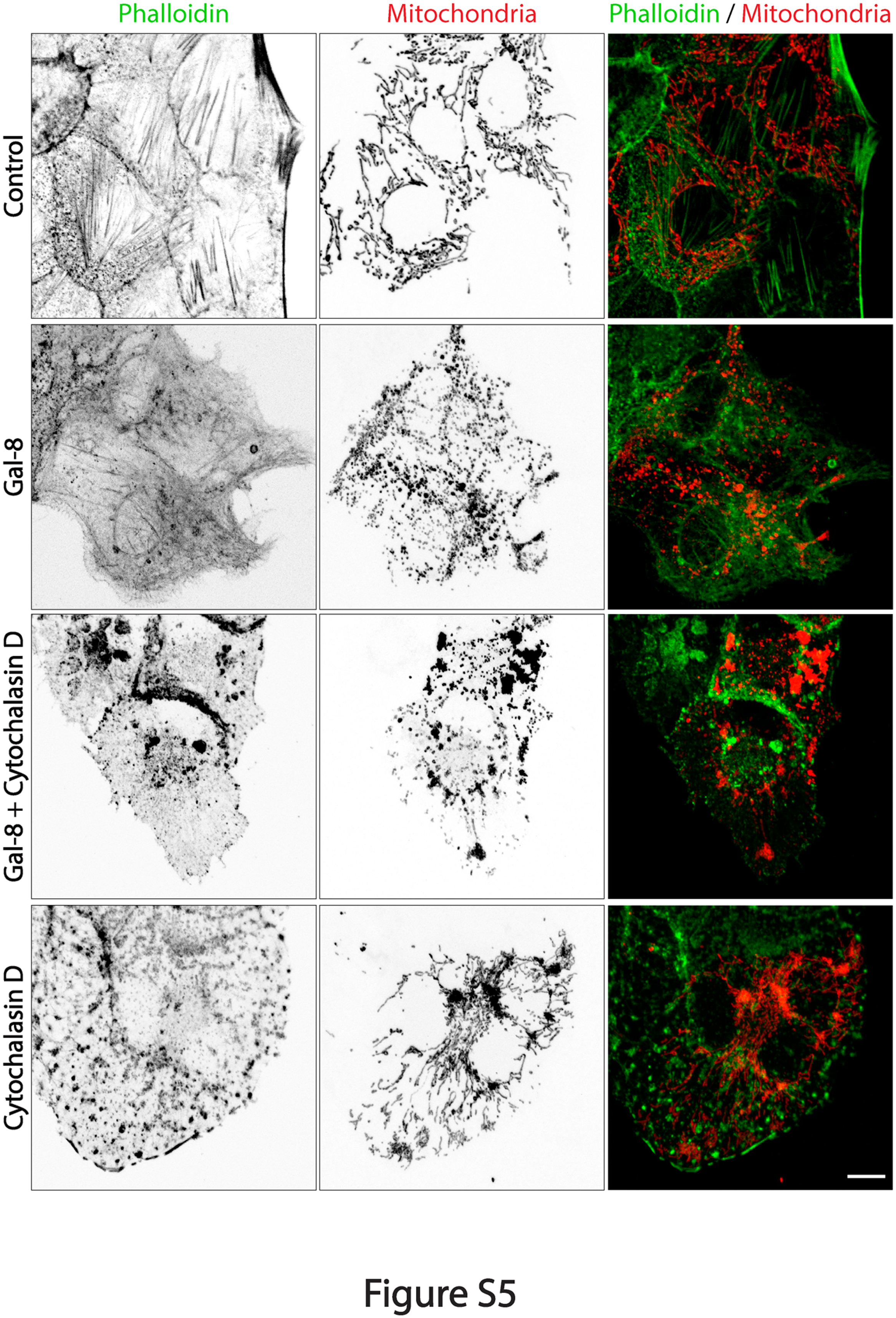

Supplement: Supplementary file 6 — Supplementary material [file mmc6.jpg]

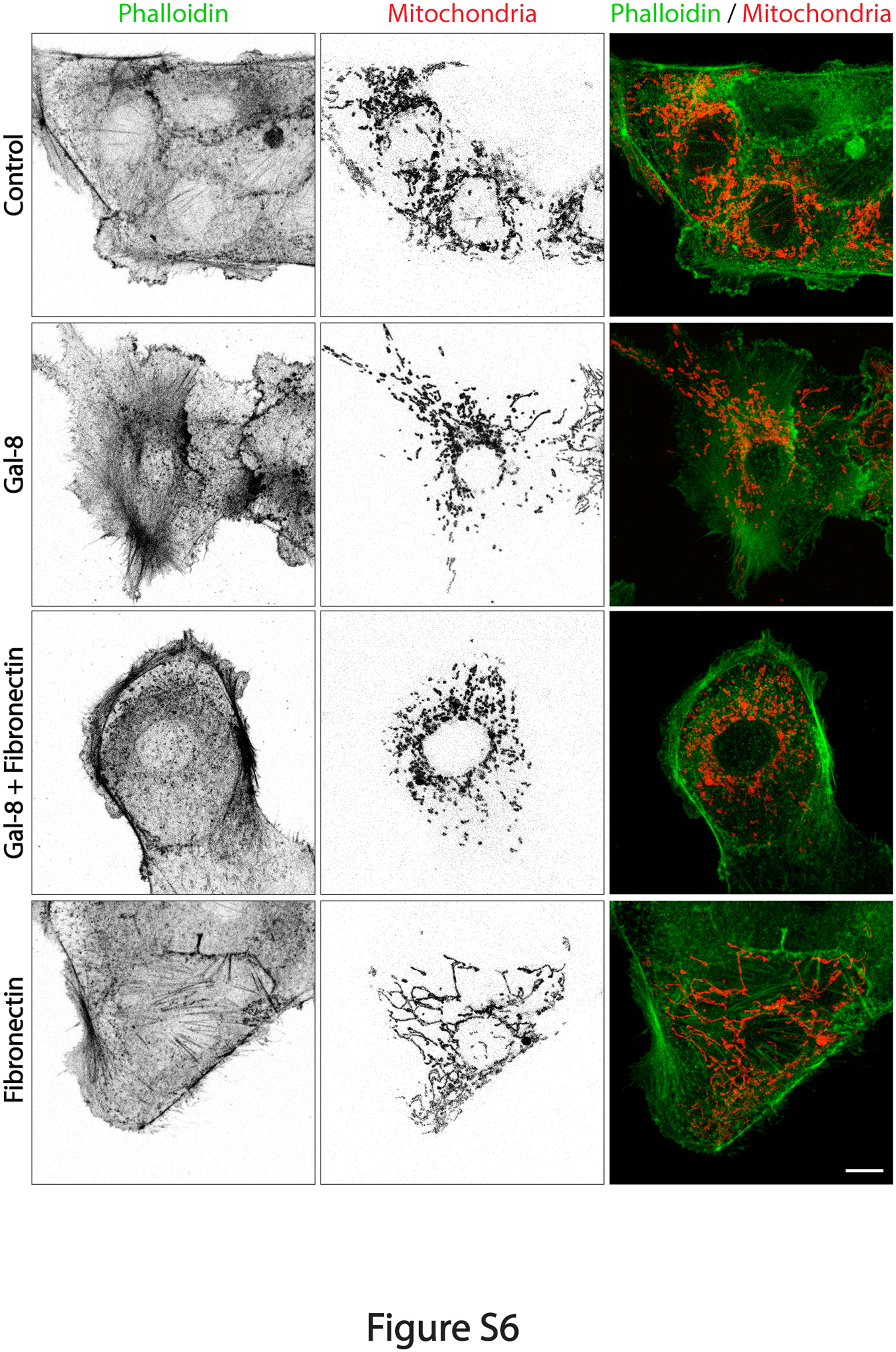

Supplement: Supplementary file 7 — Supplementary material [file mmc7.jpg]
